# Correction: Genetic algorithm-based personalized models of human cardiac action potential

**DOI:** 10.1371/journal.pone.0244687

**Published:** 2020-12-22

**Authors:** Dmitrii Smirnov, Andrey Pikunov, Roman Syunyaev, Ruslan Deviatiiarov, Oleg Gusev, Kedar Aras, Anna Gams, Aaron Koppel, Igor R. Efimov

The Data Availability statement for this paper is incorrect. The correct statement is: All optical mapping, CAGE and RNA-seq data used in the study are available using the following DOI: https://doi.org/10.5061/dryad.stqjq2c09.
