# Supplementary figures and images for: Genetic algorithm-based personalized models of human cardiac action potential
Source: PLoS One. 2020 May 11;15(5):e0231695. doi: 10.1371/journal.pone.0231695 (PMC7213718; doi:10.1371/journal.pone.0231695)

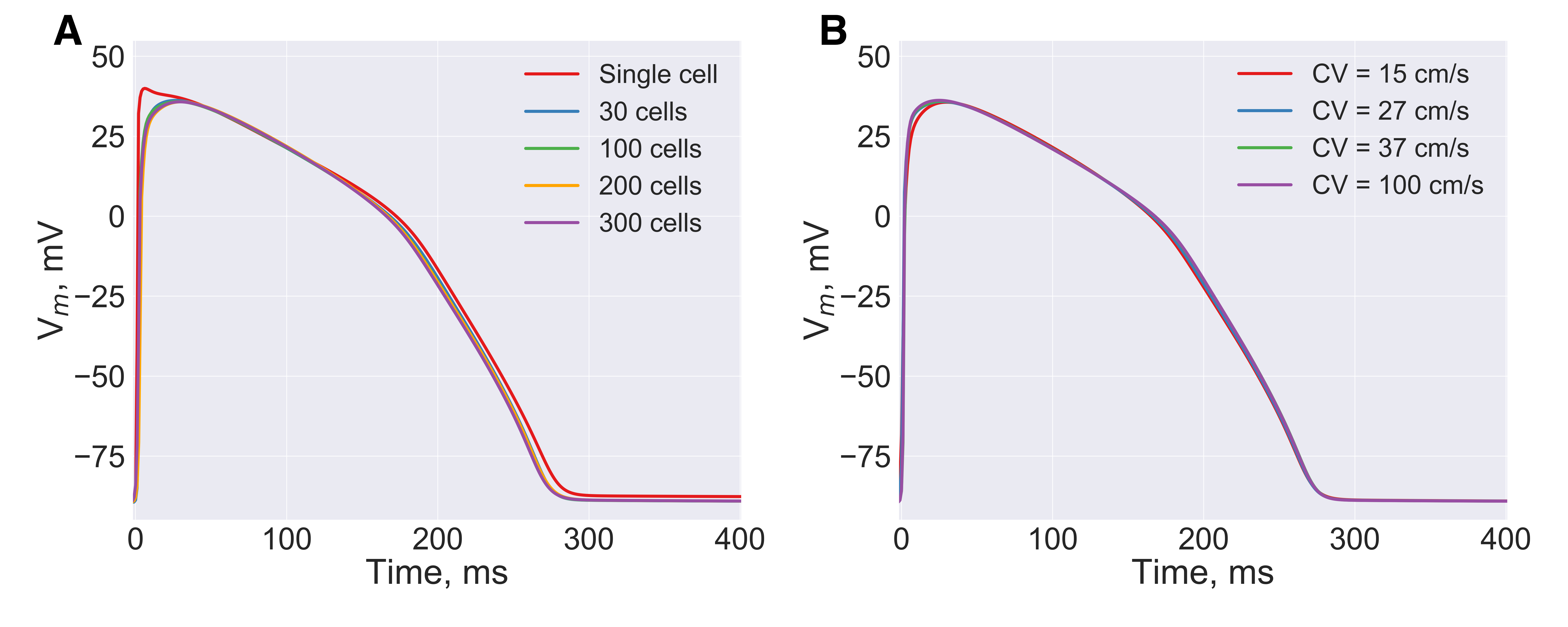

Supplement: S1 Fig — (A) Comparison of a single cell AP waveform (red line) and AP waveforms recorded from a central cell of a 1D string of cells, tissue size was varied, CV was ≈27 cm/s in tissue simulations. (B) Comparison of AP waveforms recorded from a central cell of a 100-cells long (1 cm) string of cells with variable gap junctions conductivity. (TIFF) [file pone.0231695.s001.tiff]

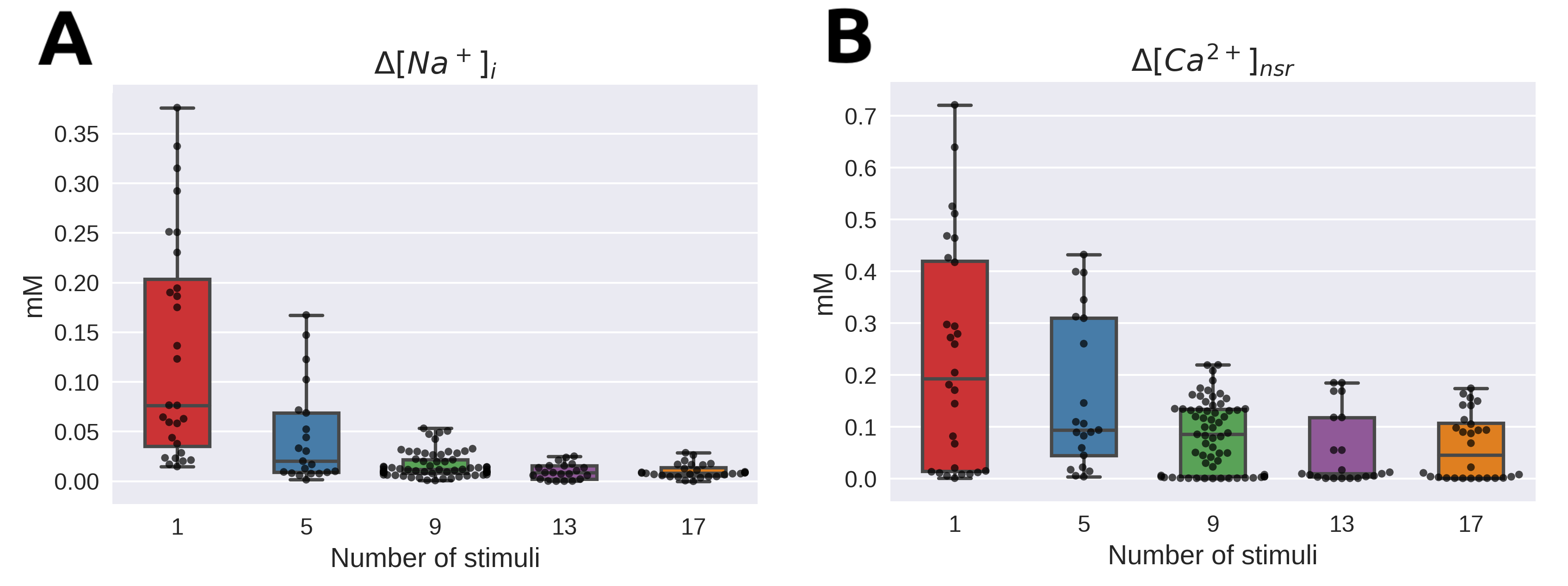

Supplement: S5 Fig — (TIFF) [file pone.0231695.s005.tiff]

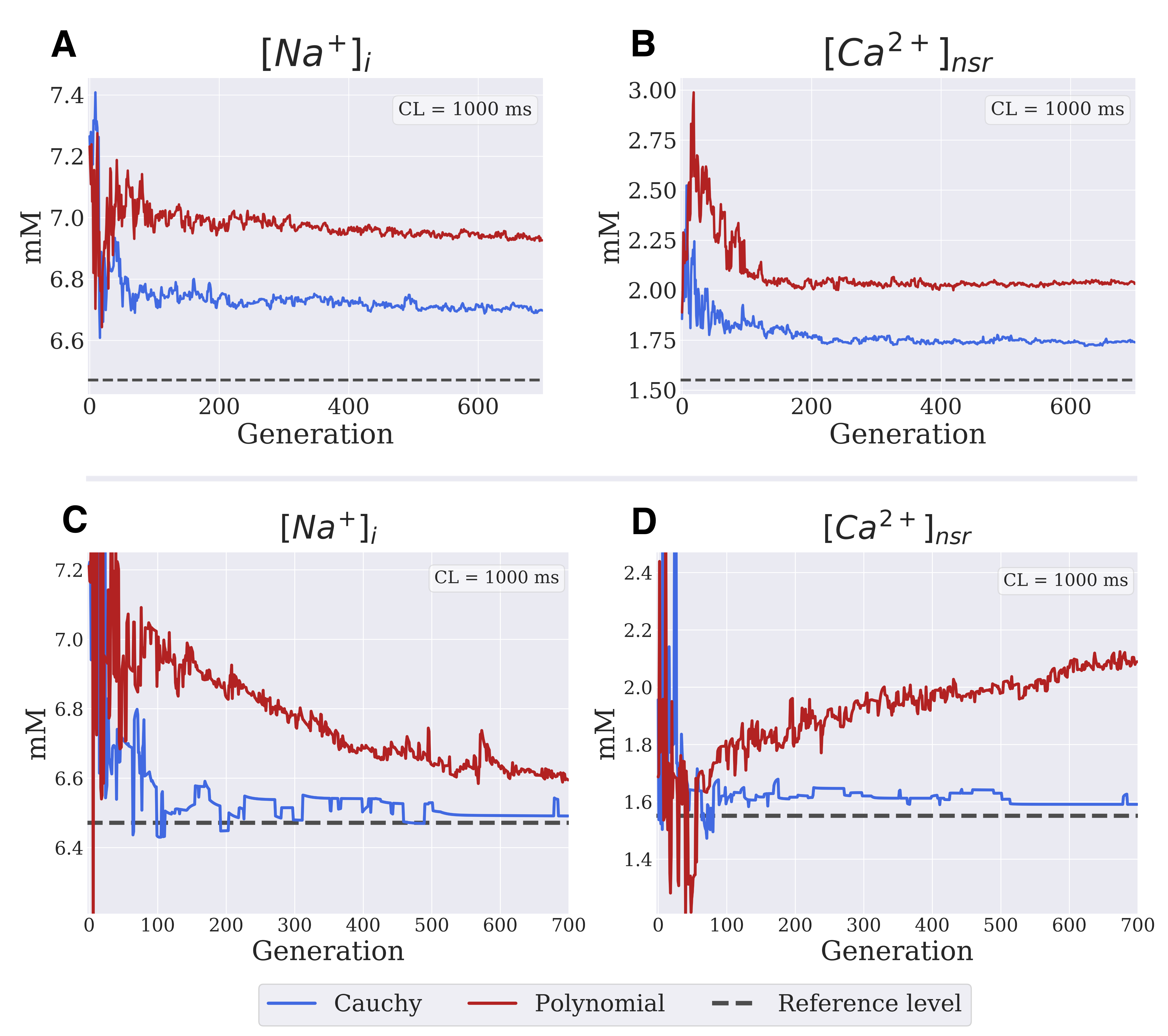

Supplement: S6 Fig — (A, B) Best organism intracellular [Na+]i and [Ca2+]nsr concentrations averaged over 9 GA runs plotted against generation number. Dashed line in both panels corresponds to input model concentration values. (C, D) Intracellular [Na+]i and [Ca2+]nsr concentrations taken from one of the GA runs. (TIFF) [file pone.0231695.s006.tiff]

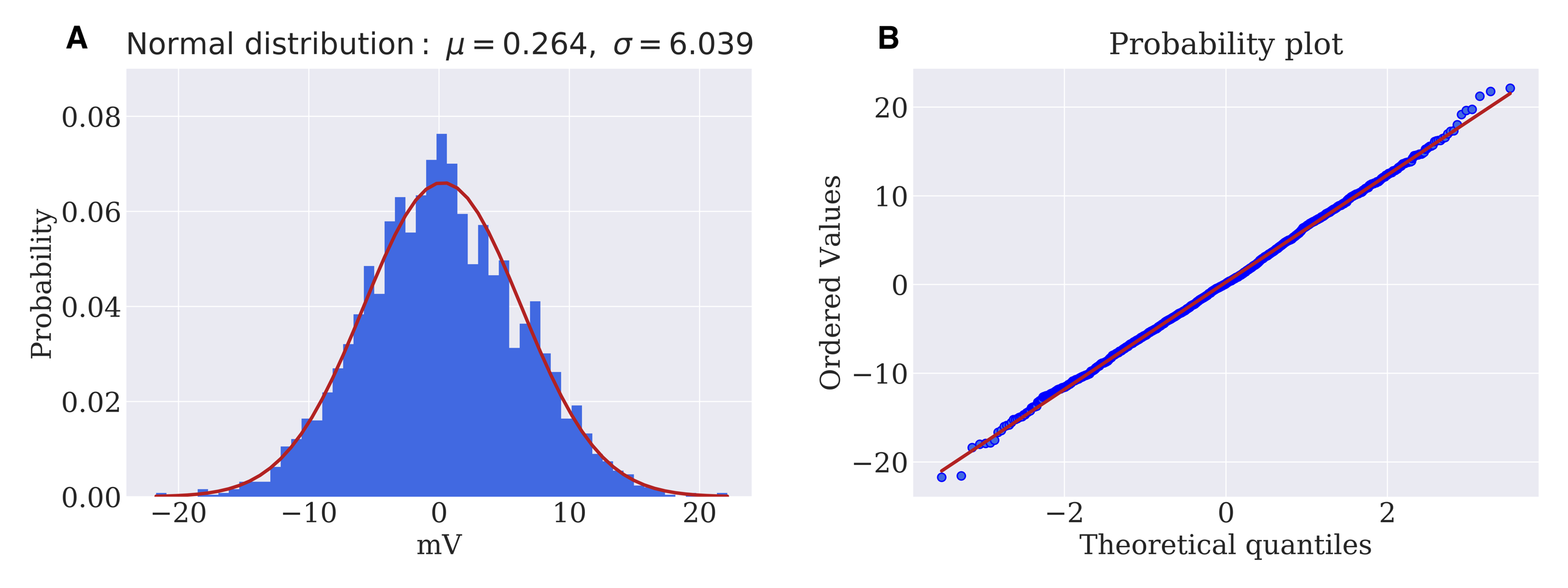

Supplement: S12 Fig — (A) Experimental noise is reproduced the normal distribution with mean = 0.264 mV, and standard deviation = 6.039 mV. (B) Corresponding probability plot: quantiles of experimental noise amplitude distribution (blue) are plotted against quantiles of a theoretical normal distribution (red line). (TIFF) [file pone.0231695.s012.tiff]

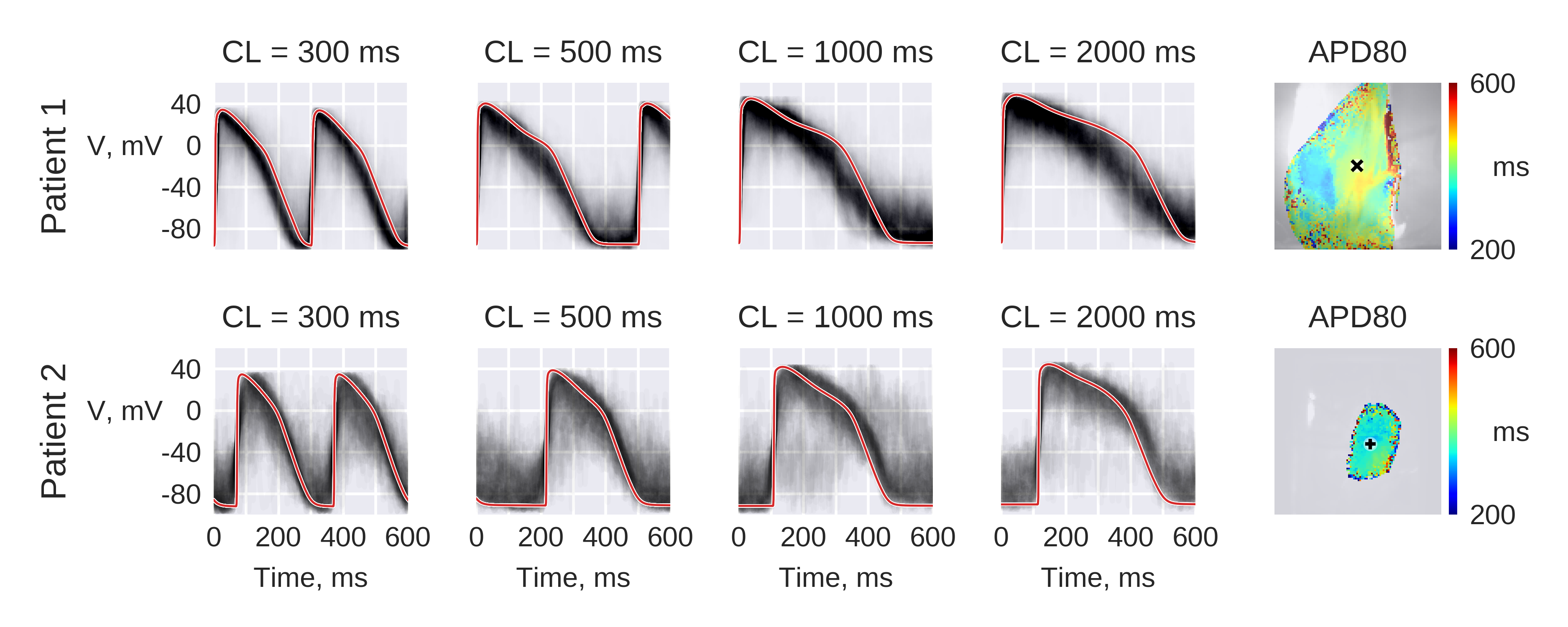

Supplement: S13 Fig — Grey lines depict AP waveforms recorded from the wedge preparations. Experimental waveforms are aligned to match the time corresponding to (dV/dt)MAX. Red lines correspond to Patient 1 GA-output model (top row) and Patient 2 mRNA-based model (bottom row). The pixels of AP waveform recording that was used as input to GA is marked by the “x” symbol on the top APD map. The pixel of the recording that was used on Fig 9 to compare Patient 2 model with experimental AP is marked by the “+” symbol on the bottom APD maps. (TIFF) [file pone.0231695.s013.tiff]
