# Supplementary material for: Genetic algorithm-based personalized models of human cardiac action potential
Source: PLoS One. 2020 May 11;15(5):e0231695. doi: 10.1371/journal.pone.0231695 (PMC7213718; doi:10.1371/journal.pone.0231695)
Supplement: S1 Text — (PDF) [file pone.0231695.s014.pdf]

SUPPLEMENTARY MATERIALS

Source code

The current version of the presented Genetic Algorithm implementation is available online at: https://github.com/humanphysiologylab/Genetic-Algorithm

Tissue simulation.

The algorithm developed in the current study was optimized to reconstruct model parameters using optical mapping action potential (AP) recordings in the cardiac tissue. Intercellular interactions affect AP waveform; therefore, 1D tissue was simulated to evaluate the fitness function. For each pacing cycle length (PCL) the following system of ordinary differential equations was solved:

$$\frac{dV_m}{dt} = -\frac{1}{C_m}\sum_i I_i + I_{gap} = -\frac{1}{C_m}\sum_i I_i + g_{gap}(V_{m-1} - 2V_m + V_{m+1}),$$

where V is the membrane potential, C_m – cell membrane capacitance, I_i – transmembrane ionic currents, I_{gap} – junctional current, g_{gap} – gap junctions' conductivity, V_{m+1} and V_{m-1} are downstream and upstream cells, the size of a cell taken to be *100 µm*.

We have found that in case of relatively low conduction velocity (CV) value of *27 cm/s*, 30 cells-long (*3 mm*) tissue is adequate to minimize the boundary effects on the AP waveform (S1A Fig). On the other hand, 100-cells (*1 cm*) long tissue simulations demonstrate that within physiological range (*20-100 cm/s*) exact CV value does not affect exact AP waveform (S1B Fig). Therefore, a combination of *3 mm* long tissue size and *27 cm/s* CV was used in all genetic algorithm (GA) runs in this study.

Input data requirements: signal-to-noise ratio.

In order to test algorithm sensitivity to input AP signal-to-noise ratio (SNR), simulated input APs waveforms were distorted by Gaussian noise (S10 Fig). S11 Fig summarize output parameters sensitivity to SNR. To verify the assumption that experimental noise follows normal distribution in the optical mapping experiment, we have calculated the difference between input optical action potentials (OAP) and their corresponding GA output APs:

$$\Delta V_i = \left|V_{\exp}(i) - V_{\mathrm{mod}}(i)\right|, i = t_{start}, \ldots, t_{end},$$

where V_{exp} is experimental membrane potential, V_{mod} corresponds to model membrane potential, thus GA output AP was supposed to correspond to undistorted experimental AP. Experimental OAP were preprocessed with narrow band stop IIR Butterworth filter to remove 60 Hz-hum, furthermore, depolarization phase was excluded from the set (to exclude photon scattering effects from the consideration). As demonstrated by histogram and probability plot on S12 Fig experimental noise is indeed close to normal distribution.

Table A: CAGE expression levels relative to *Patient 1.*

	Patient 1	Patient 2	Patient 3	Patient 4	Patient 5	Patient 6	Patient 7
KCNJ2 (IK1)	1	1.127	0.965	0.721	0.757	0.949	1.104
KCNH2 (IKr)	1	1.401	1.214	1.431	1.998	1.565	3.064
KCNQ1 (IKs)	1	0.803	0.465	0.759	0.578	0.719	0.745
SCN5A (INa)	1	0.901	0.707	0.557	0.583	0.658	1.198
KCNA4 (Ito)	1	0.919	0.66	0.916	0.996	0.848	1.233
CACNA1C (ICaL)	1	0.973	1.019	0.904	1.256	1.363	1.463
SLC8A1 (INCX)	1	1.025	1.538	0.864	5.347	1.959	2.079
ATP1A1 (INaK)	1	0.931	0.642	0.902	0.643	0.751	0.802
ATP2B4 (IpCa)	1	0.673	1.005	0.461	1.998	0.902	2.649
ATP2A2 (SERCA)	1	0.769	0.878	0.754	0.153	0.444	0.649
CALM1 (CaM)	1	0.889	0.589	0.67	0.645	0.653	0.535
RYR2 (RyR)	1	1.06	0.926	0.941	0.772	0.987	0.573
CAMK2D (CaMKII)	1	0.776	0.89	0.811	0.789	1.012	1.979

Table B: mRNA-seq expression levels relative to *Patient 8.*

	Patient 8	Patient 9	Patient 10	Patient 11	Patient 12	Patient 13	Patient 14
KCNJ2 (IK1)	1	1.664	1.419	1.155	1.349	0.851	1.508
KCNH2 (IKr)	1	1.003	0.739	1.143	1.112	1.124	0.79
KCNQ1 (IKs)	1	0.868	0.726	0.885	1.041	0.846	0.822
SCN5A (INa)	1	0.86	0.802	1.145	0.828	0.837	1.015
KCNA4 (Ito)	1	1.301	2.343	2.811	1.632	2.617	2.552
CACNA1C (ICaL)	1	0.858	0.938	0.902	0.919	0.953	1.086
SLC8A1 (INCX)	1	1.417	1.048	1.199	0.995	0.673	1.112
ATP1A1 (INaK)	1	1.31	0.987	1.015	1.063	0.626	0.942
ATP2B4 (IpCa)	1	0.829	1.287	0.88	0.759	0.733	0.788
ATP2A2 (SERCA)	1	0.845	0.932	0.991	1.019	0.654	0.66
CALM1 (CaM)	1	1.127	1.021	1.073	1.068	1.079	0.958
RYR2 (RyR)	1	0.879	1.095	0.992	0.995	0.95	0.914
CAMK2D (CaMKII)	1	0.867	0.991	1.096	0.894	0.94	1.139

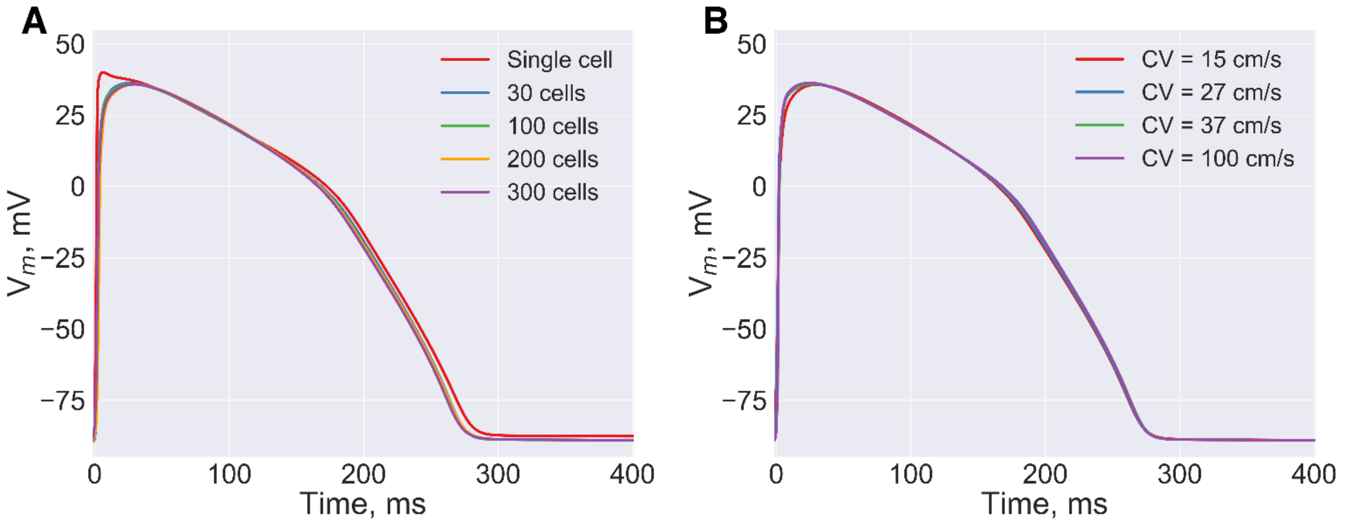

S1 Fig. Tissue effects.

(A) Comparison of a single cell AP waveform (red line) and AP waveforms recorded from a central cell of a 1D string of cells, tissue size was varied, CV was ≈*27 cm/s* in tissue simulations. (B) Comparison of AP waveforms recorded from a central cell of a 100-cells long (*1 cm*) string of cells with variable gap junctions conductivity.

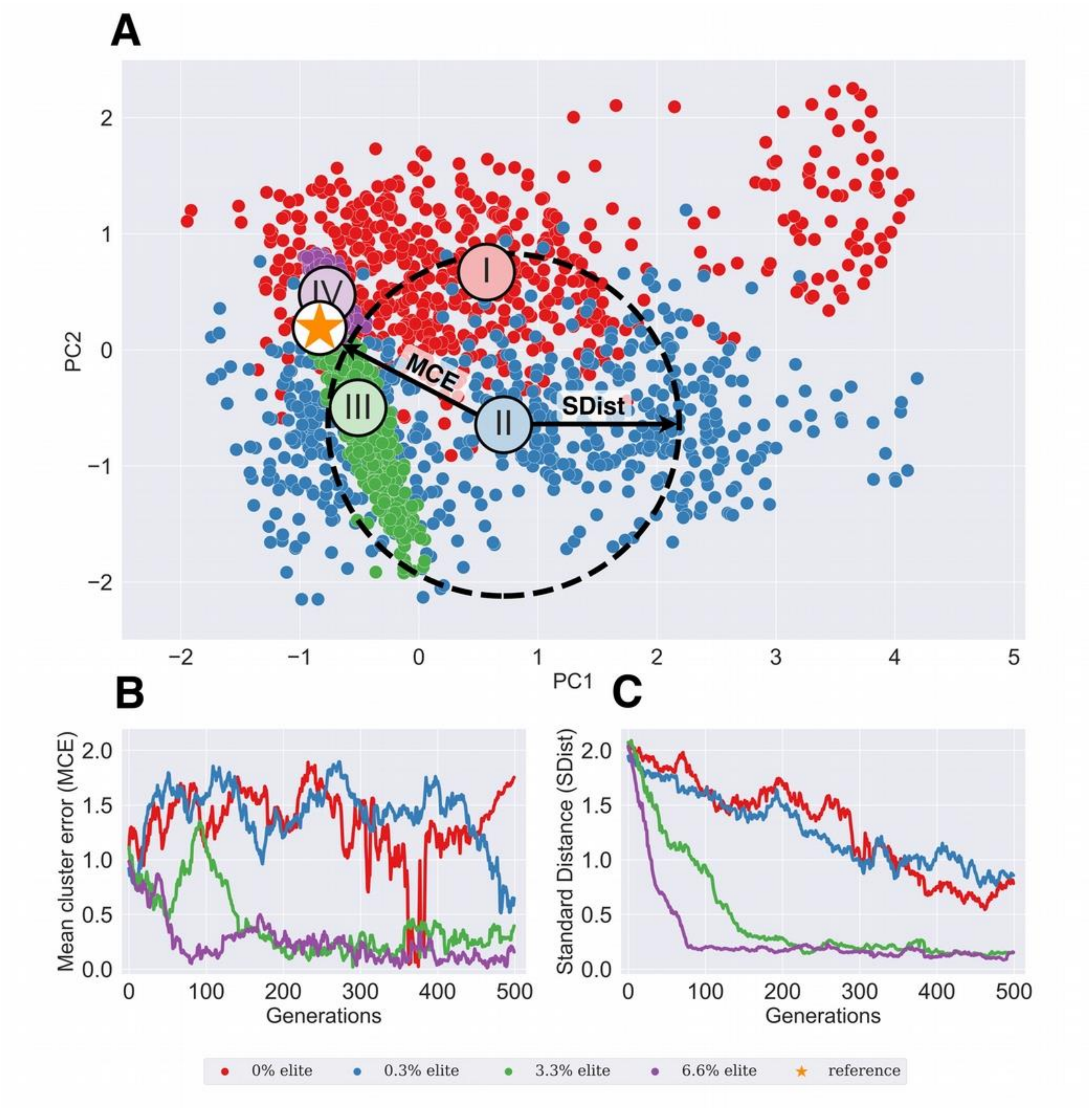

S2 Fig. Clusters characteristics: Mean Cluster Error (MCE) and Standard Distance (SDist). (A) Mean cluster error (distance between the center of each cluster and reference value) and Standard Distance (plotted as a radius of dashed circle, measures the size of a distribution). Cluster mean centers are shown by numbers I (corresponding to 0% of elite organism, red points), II (0.3% of elite organism, blue points), III (3.3% of elite organisms, green points), IV (6.6% of elite organisms, purple points). (B) MCE dependence on generation number for each cluster. Purple and green clusters rapidly shift to the exact solution neighborhood and remain there until the GA termination, while red and blue clusters don't converge to the reference value. (C) SDist dependence on generation number for each cluster. Purple cluster size decreased approximately 8 times after a hundred of generations. Red and blue clusters size decreased 2.6 times after 500 generations.

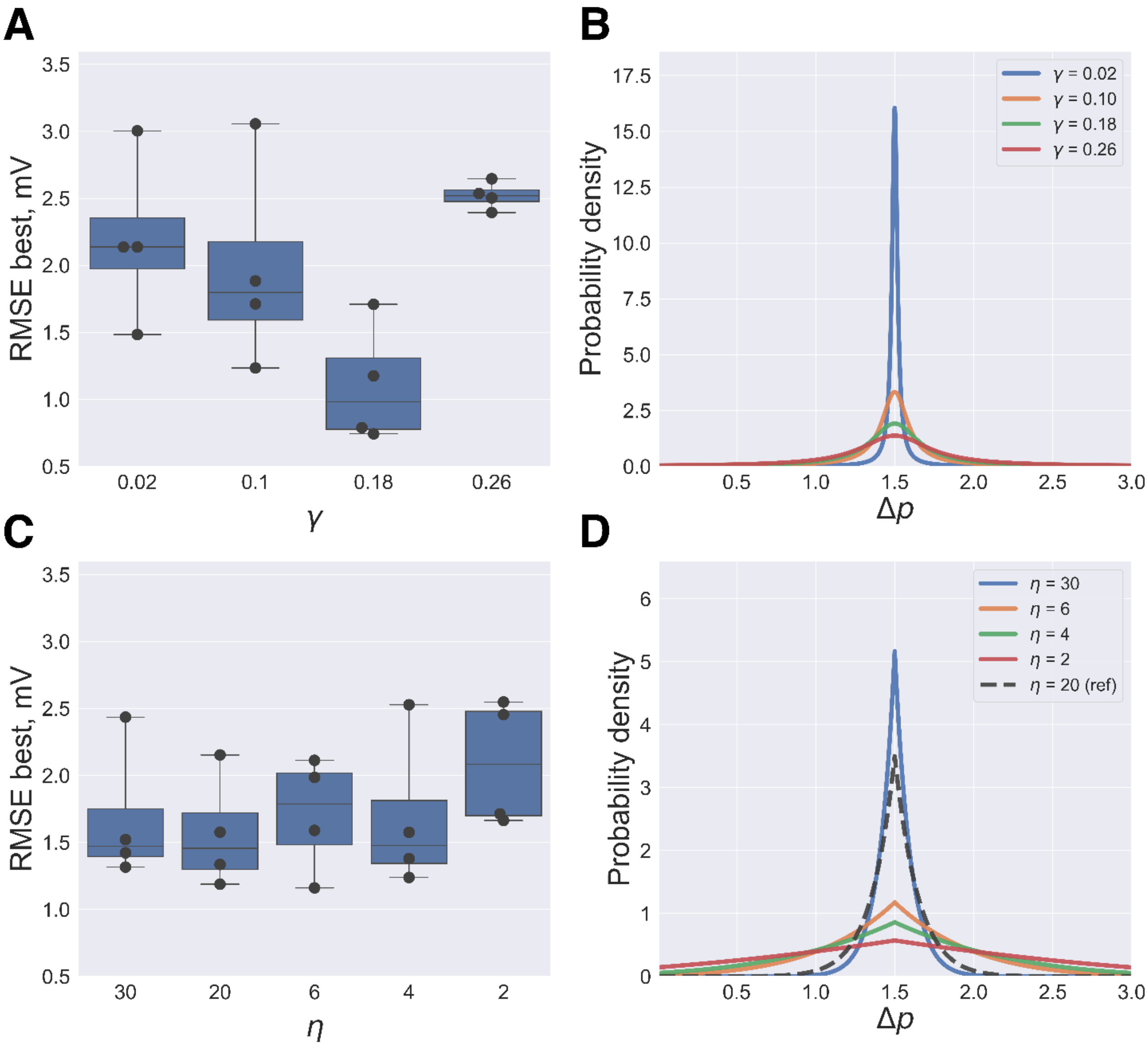

S3 Fig. Polynomial and Cauchy mutations with different distribution parameters. (A) Best organism RMSE dependence on the γ parameter of the Cauchy distribution on generation № 700. **(B)** Cauchy distribution probability density function dependence on the γ parameter. **(C)** Best organism RMSE dependence on the η parameter of the polynomial distribution on generation № 700. **(D)** Polynomial distribution probability density function dependence on the η parameter.

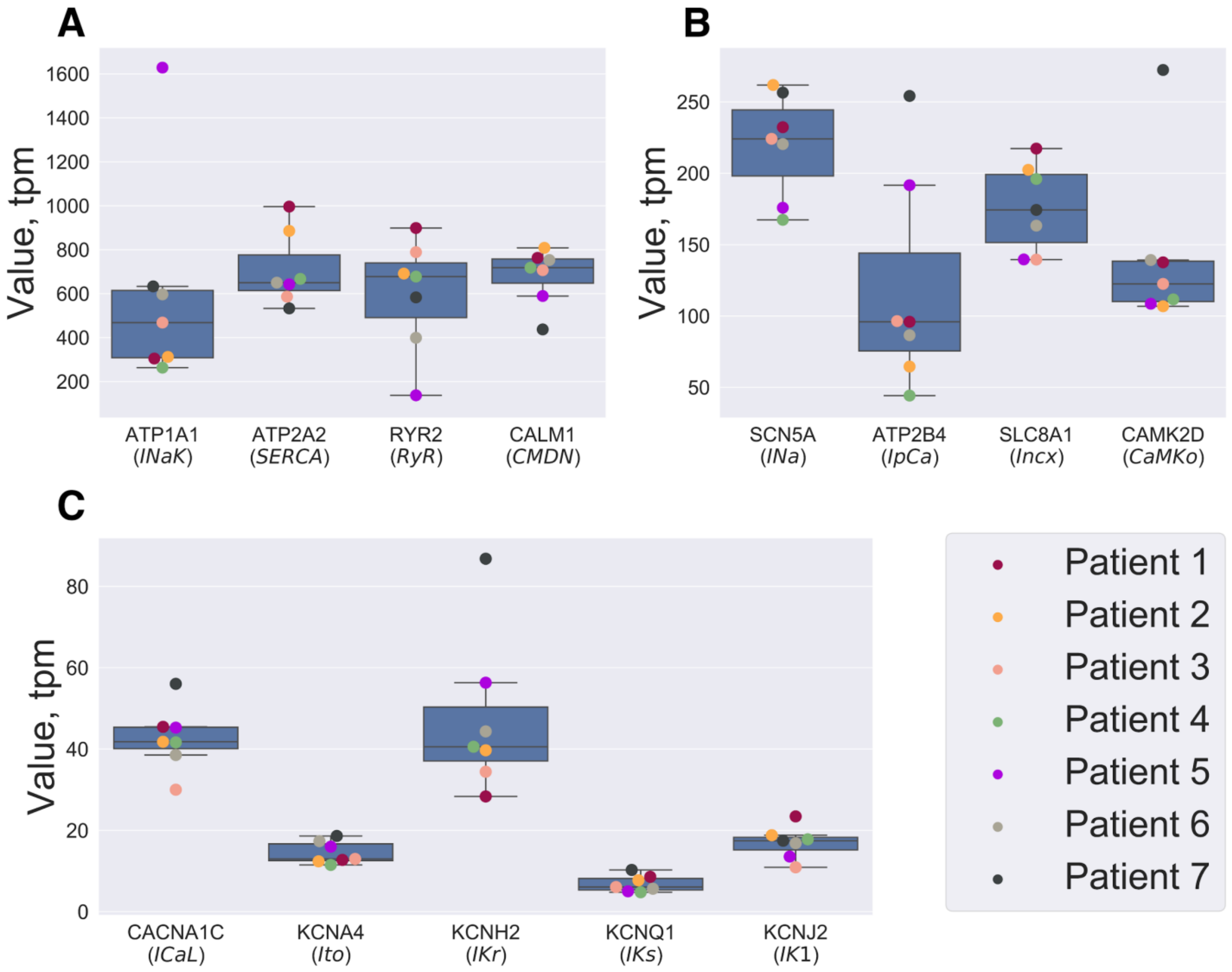

S4 Fig. CAGE measured mRNA-expression profiles for Patients 1 -- 7. The mRNA expression level measured in 7 donor hearts. Only genes used for rescaling model parameters are shown. Outliers were determined by IQR method. Colors correspond to APs and restitution curves shown in Fig. 9.

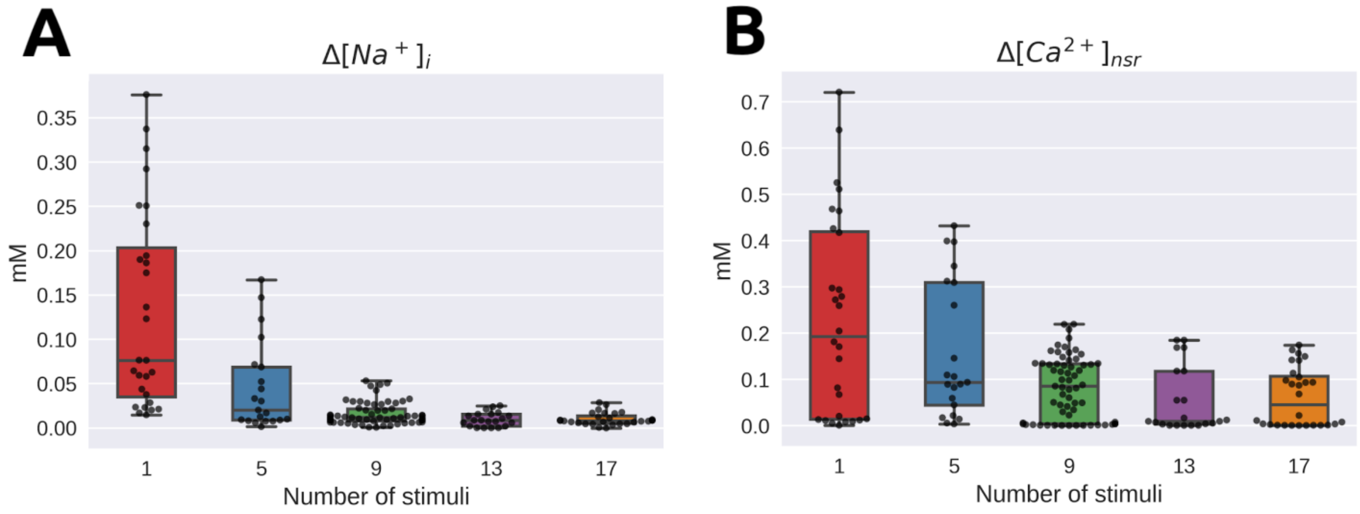

S5 Fig. $[Na^+]_i$ and $[Ca^{2+}]_{nsr}$ distance from steady-state values dependence on number of beats per generation.

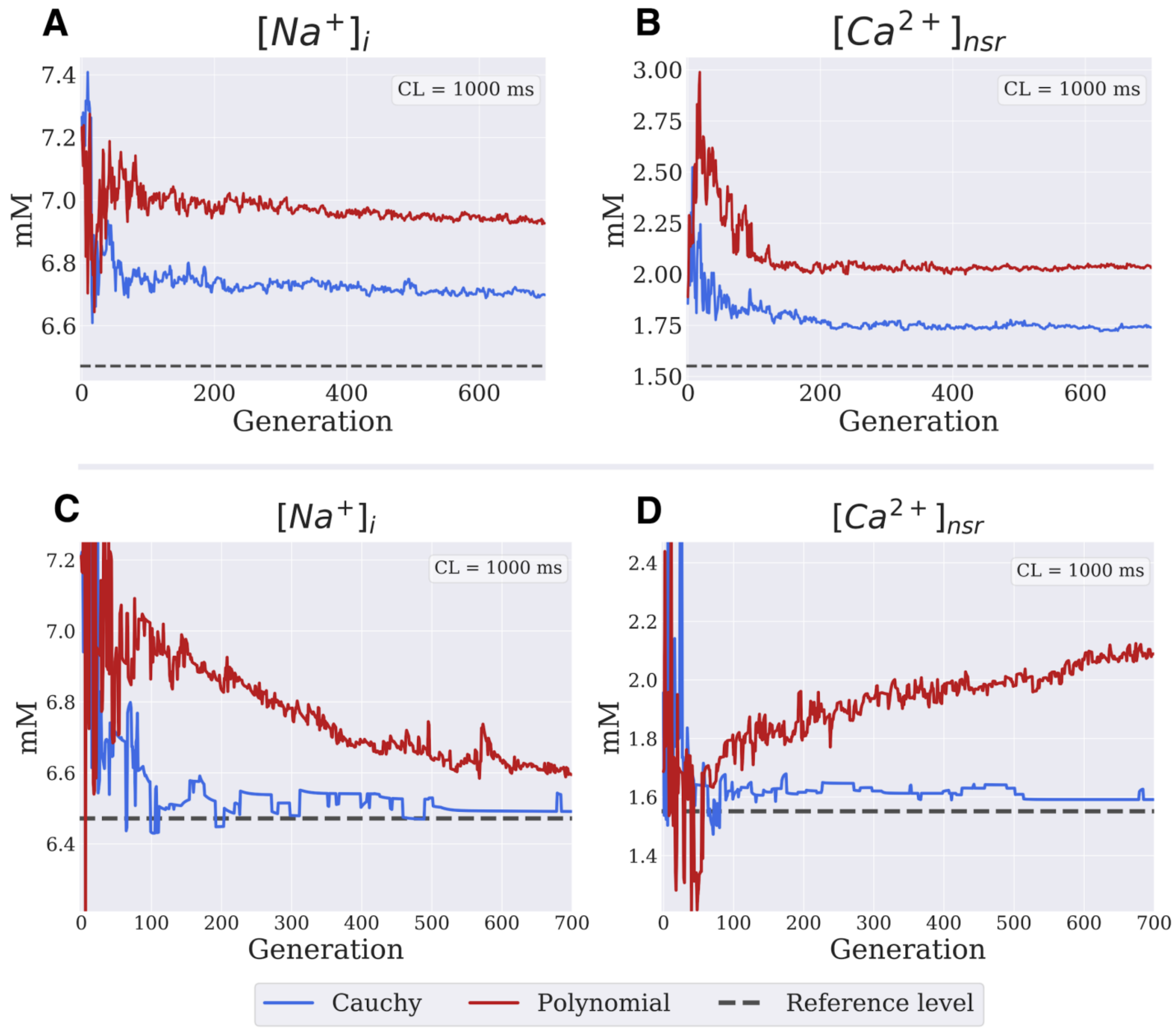

S6 Fig. Dynamics of $[Na^+]_i$ and $[Ca^{2+}]_{nsr}$ concentration. (A,B) Best organism intracellular $[Na^+]_i$ and $[Ca^{2+}]_{nsr}$ concentrations averaged over 9 GA runs plotted against generation number. Dashed line in both panels corresponds to input model concentration values. (C,D) Intracellular $[Na^+]_i$ and $[Ca^{2+}]_{nsr}$ concentrations taken from one of GA runs.

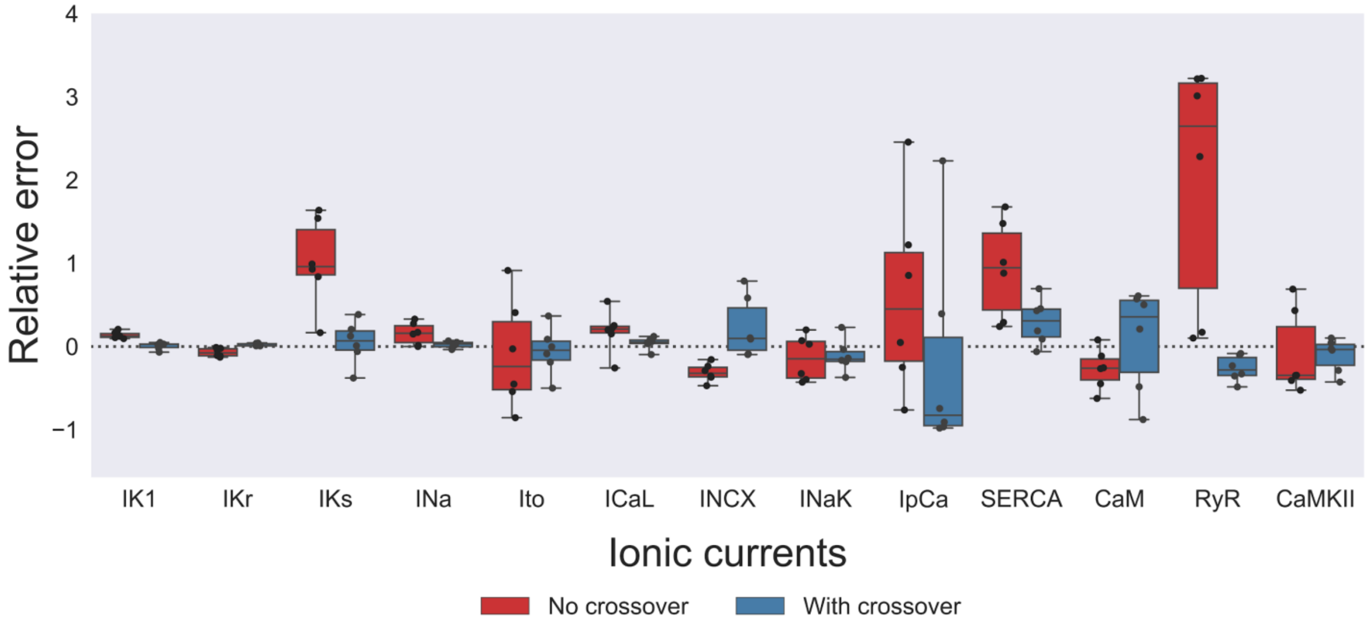

S7 Fig. Convergence without crossover. Best organism parameter values on generation № 700 of GA runs with (blue boxes, n=6) and without (red boxes, n=6) crossover operator.

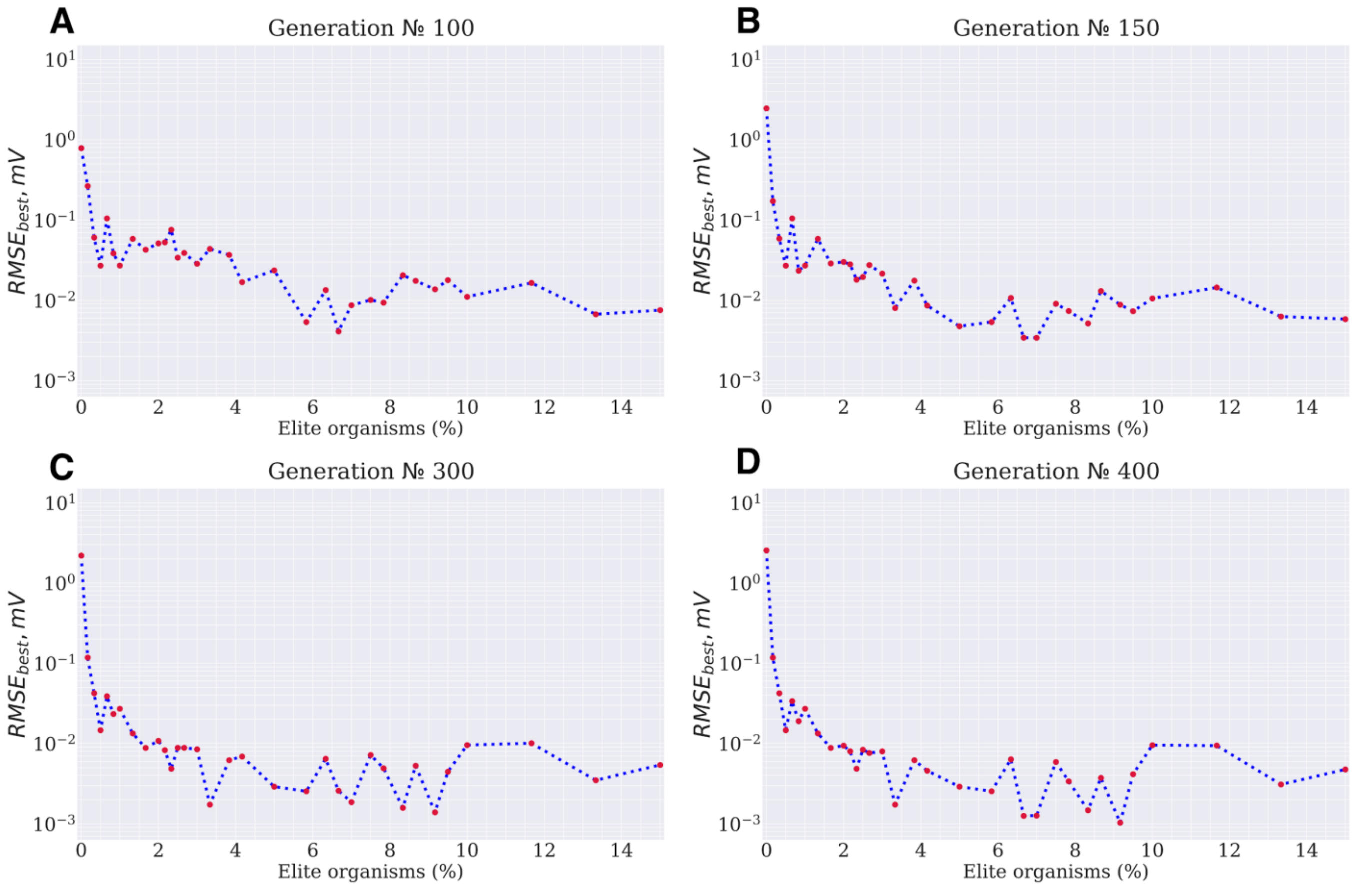

S8 Fig. Best organism RMSE dependence on the number of elite organisms.

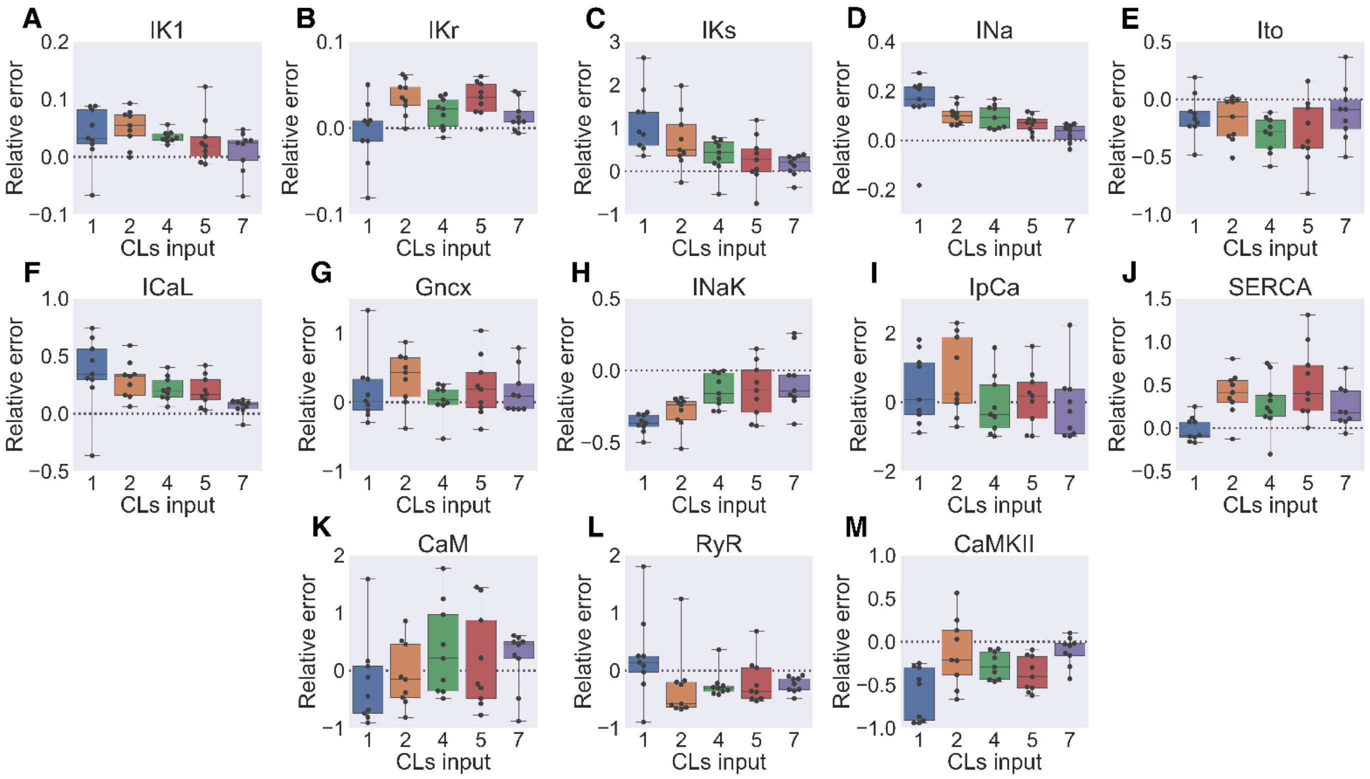

S9 Fig. Solution sensitivity to the number of input baselines.
(A-M) Box-and-whiskers plots depict the model parameters sensitivity to the number of input AP baselines. Input AP was simulated at several PCLs listed in the Fig 7A. Dashed line corresponds to the input model parameter value.

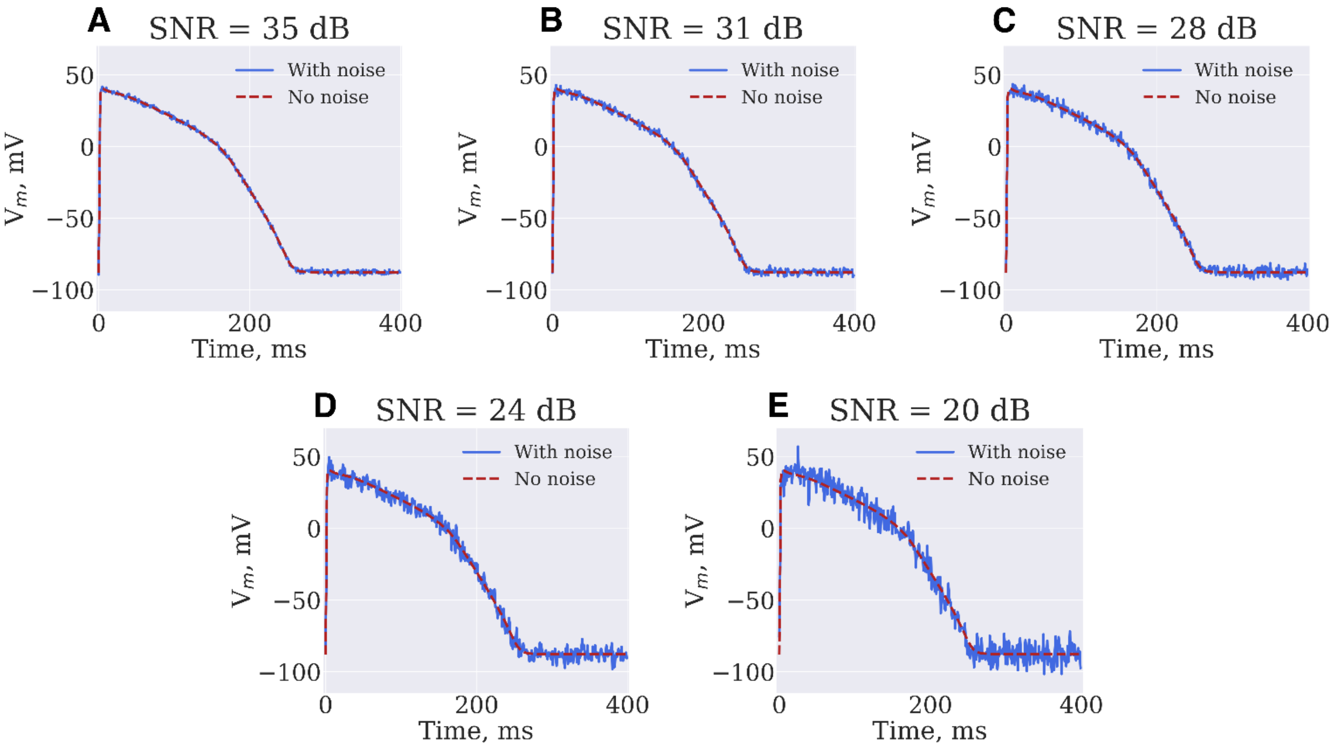

S10 Fig. Input baselines signal-to-noise ratio.
(A-E) APs waveforms (blue curves) for the different SNR values: 35 dB, 31 dB, 28 dB, 24 dB, 20 dB. Red dashed lines correspond to precise signal with CL = 1000 ms.

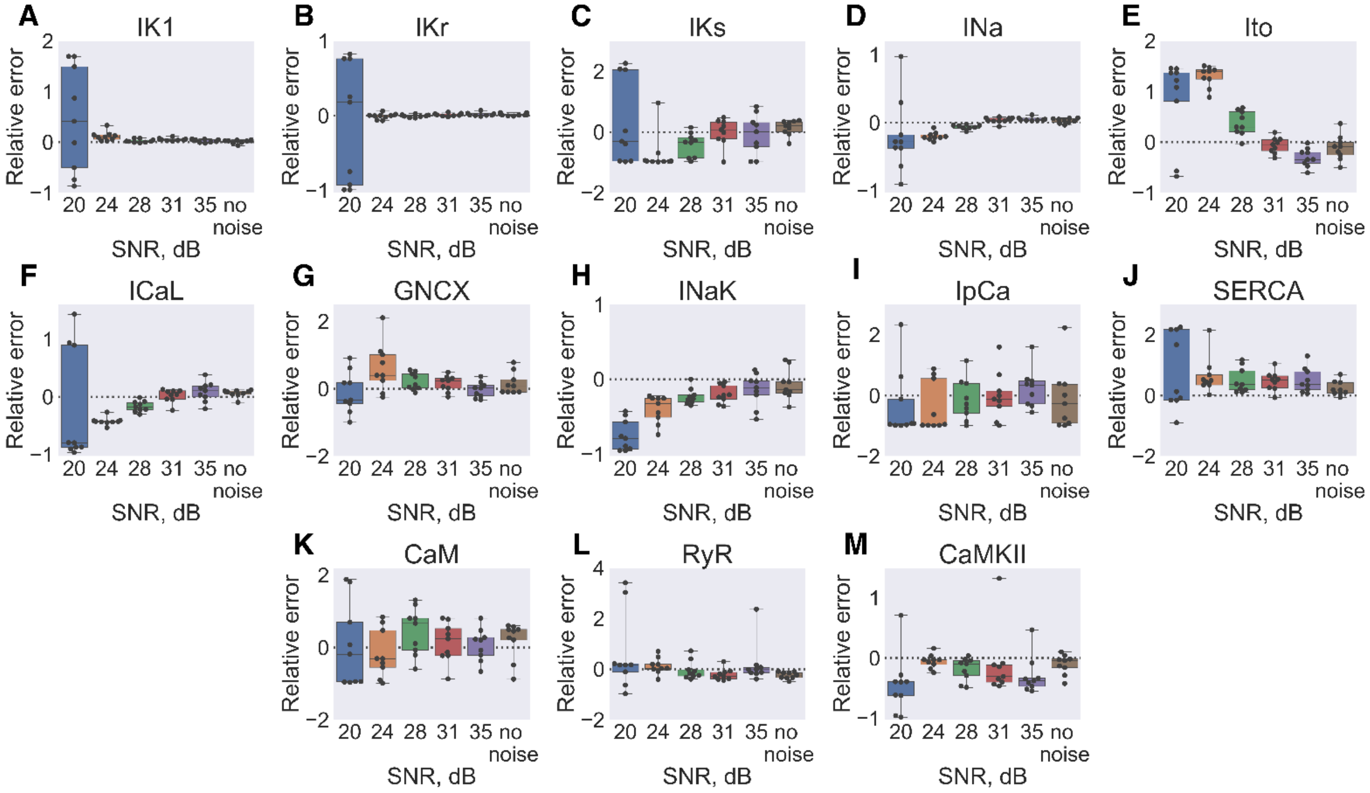

S11 Fig. Parameters dependence on the SNR.

(A-M) Optimized model parameters distribution depending on the SNR of input APs. Dashed line depicts input model parameter value.

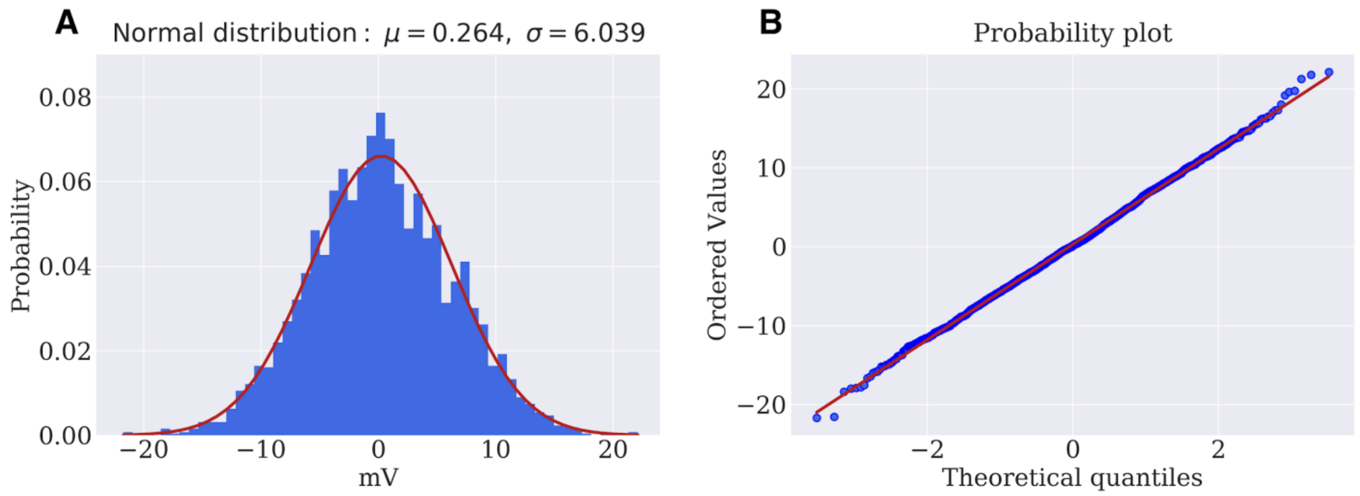

S12 Fig. Gaussian noise.
(A) Experimental noise is reproduced the normal distribution with mean = 0.264 mV, and standard deviation = 6.039 mV. (B) Corresponding probability plot: quantiles of experimental noise amplitude distribution (blue) are plotted against quantiles of a theoretical normal distribution (red line).

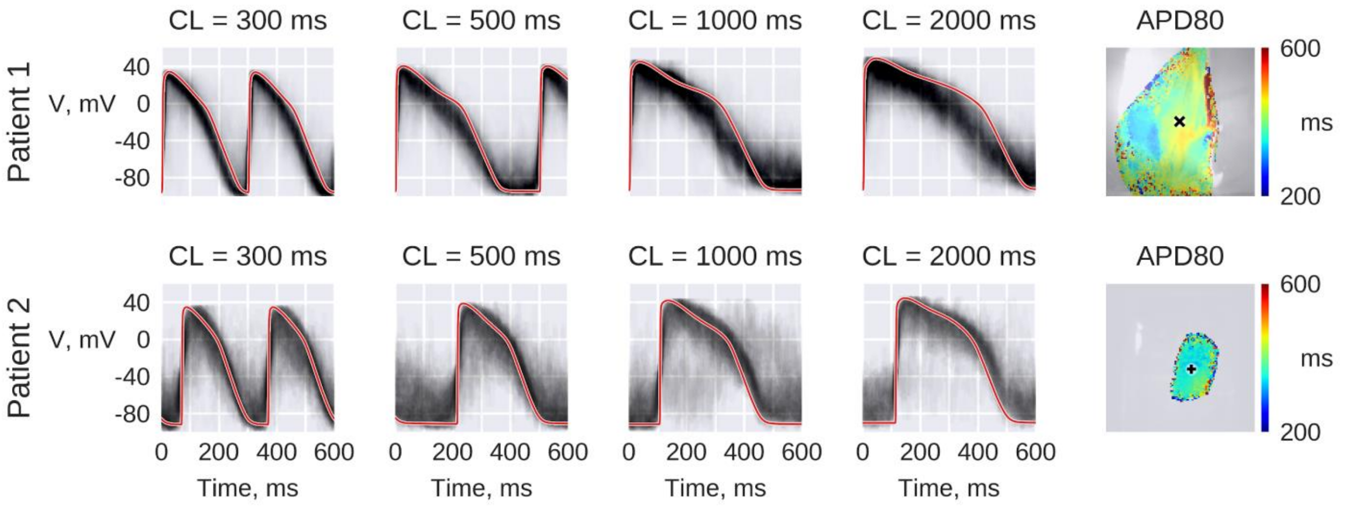

S13 Fig. Heterogeneity of APD for Patient 1 and Patient 2. Grey lines depict AP waveforms recorded from the wedge preparations. Experimental waveforms are aligned to match the time corresponding to $(dV/dt)_{MAX}$. Red lines correspond to *Patient 1* GA-output model (top row) and *Patient 2* mRNA-based model (bottom row). The pixels of AP waveform recording that was used as input to GA is marked by the “x” symbol on the top APD map. The pixel of the recording that was used on Fig. 9 to compare *Patient 2* model with experimental AP is marked by the “+” symbol on the bottom APD maps.
