# Supplementary material for: Genetic algorithm-based personalized models of human cardiac action potential
Source: PLoS One. 2020 May 11;15(5):e0231695. doi: 10.1371/journal.pone.0231695 (PMC7213718; doi:10.1371/journal.pone.0231695)
Supplement: Attachment — Submitted filename: Reviewers_responce.docx [file pone.0231695.s015.docx]

We sincerely thank both reviewers for the time and effort they put into very extensive reviews and insightful critiques to the manuscript. Their critiques served us as a welcome guide during extensive revisions and additional research. We apologize that it took us long time to address their concerns, but it was due to very extensive nature of the critiques. In reply to the reviewers’ questions we ran additional simulations and thoroughly revised the text. Both reviewers mentioned that RNA-based model verification deserves much more prominence in the text. We agree with this suggestion; however, the data that we have presented in the originally submitted manuscript was not sufficient to provide a reasonable discussion of the subject. Now, in reply to reviewers concern we were able to conduct much more extensive additional research of the mRNA-based models rescaling, and we are happy to report that our new significant data provides solid support to our original premise.

We do hope that the new revision is much more comprehensible and interesting for potential reader then the initial submission. To facilitate review process, we restated original critiques in green font and provided our response in black font and new manuscript text in blue font.

***Reviewer #1:*** *Genetic algorithm-based personalized models of human cardiac action potential*

*The authors propose and implement a genetic-algorithm approach to estimate maximal conductances (and similar parameters for e.g. J_up) an action potential model, based on optical mapping of voltage in human left-ventricular preparations. Improvements to previous GA methods are presented, including a restitution based approach, and a novel mutation operator. A synthetic data study is performed, after which the method is applied to real data, and validated by comparing the obtained parameters to mRNA expression levels.*

*The work is very interesting, the focus on clinically measurable signals is novel and important, and the data from donor hearts adds further value to this study. However, the text needs major work. The manuscript is not structured well, requiring the reader to skip back and forth between sections.*

Thank you very much for positive overall assessment of our work.

*It would benefit from an introduction that clearly states the entire study's objectives (not just the GA improvement) and gives the reader a better idea of what is to come. Many of the results are stated as fact in the methods (without making clear this is something the study will attempt to prove), which further adds to the confusion.*

We thank the reviewer for bringing up this issue of the manuscript, in the revised version the manuscript introduction is extended, and states aims of the study more clearly*.*

*I believe this manuscript has the potential to be very interesting if the authors rewrite it significantly.*

*Some more work also needs to be done to show that the chosen strategy of varying part of the initial state in the optimisation still leads to a stable `steady-state'; especially in the light of the reduced number of pre-pacing beats and the interesting observation that there are multiple steady-states, with the exact state reached depending on the initial state).*

Thank you, in reply to this helpful comment we have ran significant additional simulations and paced GA output model for 1000 s to compare steady state with GA output state. The difference is shown in new Fig. 2 and corresponding text.

Major points:

*- Results section: It seems the text has been written to have the methods section between the introduction and the results. As it is now at the end of the paper, some rewriting is necessary. I am not sure why the methods are placed at the end, either, this does not seem to be the norm for PLOS ONE in the papers I checked.*

Thank you, as we have stated above the Introduction section is extended, while “Methods” section is placed in the beginning of the article.

*- Lines 120-122: Please explain why this strategy, with only 9 stimulations at each PCL, is adequate, and show data to support this claim.*

Thank you for this important comment. The strategy is explained in greater detail and a kind of proof that this technique still results in intracellular concentrations close to steady state is depicted in Fig.2 of the new version and corresponding lines 481-498 of the text. New text reads:

“Why would this technique eventually result in concentrations converging to a steady state? The rationale is the following: if given parameters vector results in an acceptable AP waveform (i.e. close to the input AP), but far from steady state, then this solution is going to be discarded eventually, since waveform is going to change after few beats. For example, if AP of particular organism on generation N minimizes RMSE, but corresponds to one of the dotted lines in Fig. 2B, then RMSE is going to be large for this particular organism on generation N+1 (one of the dashed lines).

In particular, we suggest including the initial intracellular sodium ([Na+]i) and calcium sarcoplasmic reticulum load ([Ca2+]NSR) at every PCL as model parameters. The [Ca2+]NSR concentration was chosen, because most of the calcium is stored within sarcoplasmic reticulum when the cell is at resting potential. We did not include potassium concentrations in the optimized parameters vector, since 10 mM K+i concentration changes results only in approximately 2% Nernst potential change, and thus a major concentration changes have a minor immediate effect on AP waveform. In order to test if this technique indeed results in a steady state we have run 9 GA simulations. After that, the output models on generation № 700 were paced for 1000 seconds. The observed difference between GA output state and steady state is depicted in Fig. 2E-F. The output [Na+]i was 0.016±0.012 mM from steady state, the [Ca2+]NSR was 0.08±0.07 mM from steady state.”

*- Lines 122-124: After showing that the O'Hara model has a steady-state that depends on the initial conditions, the authors vary these initial conditions at every iteration of the optimisation, and then run a limited number of repeats. It is not clear to me at all how this would help bring the system into a steady-state faster, or why this should result in a better model. Please add a more detailed explanation, and show how your approach compares to a traditional approach with longer pre-pacing periods. If this is computationally infeasible, at least show the effect of adding more beats (e.g. 5, 9, 18, and 36). I would also be interested to know if the authors consider the existence of multiple "steady states" (technically periodic orbits) as a flaw in the model or if its a realistic feature that gets 'personalized' by the GA.*

Thank you for this question! In part, we provided clarification in the reply to the previous question. Please also, find a clarification on bringing the system closer to steady state, while making “jumps” in slow variables space the lines 473-481 of the text:

“The final state variables at each PCL are saved and reused as initial state at the next generation (Fig 1A). However, given that the set of parameters minimizing RMSE is itself a function of intracellular concentrations, this approach results in an optimizer solving essentially a new problem every generation. Moreover, as shown above (Fig. 2 B-D) “fixed” initial state for the organisms should result in a “fixed” steady state that might be different from input data. The solution that we propose in this study is to perform a simultaneous search in the parametric and slow variables space, i.e. the initial values of slow variables were added to parameters vector making them susceptible to mutation and crossover operators.”

The traditional approach in some sense, was provided in the Fig 7 of the manuscript, where every organism of the original Bot et. al. algorithm was paced for 50 beats. It is computationally very expensive to provide more than 50 beats.

We have also ran additional simulations with our version of algorithm to investigate the effects of different number of beats. The results (distance from steady state) are presented on S10 Fig.

We have added additional discussion of experimental evidence of multiple steady state in lines 778-773 of the text. There is some circumstantial evidence of multiple steady states: hysteresis of APD and alternans was observed by several research groups previously. However, we cannot be sure that this phenomenon occurs in living myocytes. For example, the number of some ionic channels (IKs in particular) might be dynamically adjusted by cell in response to different factors, such as calcium stress. Consequently, there are multiple ways to explain hysteresis phenomena.

New text reads:

“We have to note here, that several groups of researchers did observe restitution hysteresis as well as alternans hysteresis [34, 35, 36]. Thus, we can hypothesize that multiple steady states is an inherent feature of a myocyte that might be personalized by the GA. On the other hand, living cardiomyocyte is a complicated dynamic system and ionic channel conductivities themselves could change over the time [37].”

*- The introduction states that modifications to GA are needed, but does not argue this very well. It would be good to mention that this is in fact one of the results shown later in the paper. Similarly, the methods section states "We have found that the original polynomial mutation tends to trap soution in the local minima", but doesn't mention that this will be shown in figure 4. The reasonining for introducing a Cauchy distribution is also not clear to me.*

Thank you, we have now mentioned this fact in the introduction. Please, see lines 113-119. New text reads:

“As we demonstrate below in the Final Algorithm subsection of the Results section, this approach results in a poor parameters convergence. We have identified two reasons behind this fact: firstly, intracellular concentrations require much more than 10 seconds to converge to a steady state; secondly, a model with the particular set of parameters may converge to different steady states depending on the initial conditions. In order to address these issues we implemented a modification of GA allowing one to perform parameters optimization and steady state search in the slow variables space simultaneously.”

Regarding the Cauchy distribution: our initial observation was that modifying the concentrations by polynomial mutation might have the following effect. The whole population prematurely converges to a single non-steady state solution. After that, even though best organism gets closer to steady state every generation (increasing RMSE), some organism from the population mutates back to the previous concentration values. Cauchy distribution reduced the probability of premature convergence and did not reduce the convergence speed at the same time. In reply to reviewers’ questions we have ran additional simulations comparing Cauchy and polynomial distributions. Although, the former on average did result in better state variables, the distance from steady state was not statistically significant on the generation № 700.

For Cauchy mutation the output [Na^+^]_i_ on the generation № 700 was 0.016±0.012 mmol from steady state, the [Ca^2+^]_NSR_ was 0.08±0.07 mmol from steady state.

For Polynomial mutation the output [Na^+^]_i_ on the generation № 700 was 0.03±0.02 mmol from steady state, the [Ca^2+^]_NSR_ was 0.10±0.12 mmol from steady state.

Therefore, we have modified the figure (Fig 6 in the new revision) and corresponding text in the lines 493-508 as follows:

“Cauchy distribution is a “pathological” distribution with infinite variance and expected value. As was noted previously [18] Cauchy mutation tends to generate offspring far away from its parent. Consequently, it prohibits algorithm stagnation in local minima and generally results in better convergence for multimodal functions. Fig 6A compares two sample runs of GA with polynomial and Cauchy mutation. In case of polynomial mutation by generation 300 the whole population converged to a vicinity of a single solution that is different from input model set of parameters (red line). We observed slow convergence on subsequent generations simultaneous with slow intracellular concentration changes (compare to S2 Fig plotting corresponding intracellular concentration changes of the best organism). In the case of Cauchy mutation every parameter except INaK converged to some vicinity of input model value by generation 300. At the same time, we did not observe algorithm stagnation: note, for example, the wide variation of IK1 and RyR parameters on generation 500. Consequently, when synthetic AP was used as input data, we observed more robust algorithm convergence in case of Cauchy mutation (Fig. 6B). For example, the error of model parameters was 14±24% vs 17±75% for IKs, 11±25% vs 45 ±23% for Ito, 6±6% vs 13 ±15% for ICaL in GA runs with Cauchy and polynomial mutation correspondingly.”

*- Lines 172-173: "as shown by Andrews curves in Fig 4C the whole population is in the immediate vicinity of a single solution". It is not clear to me how Fig 4C shows this. Figures 4D and 4E also do not look like an optimiser getting stuck in local minima.*

*- Lines 181-182: "Moreover, the best organism is unchanged for a number of generations". Please explain how choosing a different distribution to sample new mutations from has caused this effect?*

*- Lines 181-182: "the best organism is unchanged for a number of generations, allowing it to reach steady state". I'm not sure how much this figure says about the Cauchy distribution being better, and how much it says about 9 beats being too short to reach steady state. Would a Cauchy distribution still be better if you had more beats? Presumably there is some sort of trade-off between the number of beats per simulation and the number of iterations of the GA. Please explain this in greater detail.*

Thank you very much for expressing these valid concerns. As mentioned in reply to previous question we have revised this part of the text, we hope that the new Fig 6 provides better arguments in favor of Cauchy distribution.

- Lines 299-313: This is very nice work and deserves to be mentioned much more in the paper, which is almost entirely about the GA and the syntetic data study.

- Lines 551-552: "Output model parameters ... between two patients.". Please explain this in much greater detail, here in the methods section, but also in the introduction or in the relevant part of the results section.

Thank you for a nice comment! We have conducted additional experiments and extended the corresponding sections extensively to address your questions.

Minor points

*- The authors present a novel adatation of a genetic algorithm, but do not present any arguments why genetic algorithms are suitable optimisers in this case. I would suggest adding either a comparison to other optimisers (e.g. CMA-ES), or at least provide some arguments why other methods are unsuitable and a custom GA is required.*

We have added additional discussion of the subject in the lines 806-815. Although it would be interesting to compare different optimizer, some custom modification would be required to find the steady-state solution. This kind of benchmarking would be very complicated and we believe it to be beyond the scope of current study. But we have modified the text as follows:

“It is worth noting that all these modifications to GA essentially make the algorithm similar to particle-based algorithms. For example, multivariate jumps are used by Particle Swarm [38], Covariance Matrix Adaptation Evolution Strategy [39] and Cuckoo Flight [40] algorithms. The last one uses “pathological” distribution similar to Cauchy mutation for Levy-flight walk. Although it would be very interesting to compare these algorithms performance with GA modification proposed in this study, some custom modifications to the aforementioned algorithms are still required: as was noted above same AP waveform could be reproduced by different parameter sets [8]. In order to narrow down the output parameters range, not steady state solutions should be somehow automatically discarded by the algorithm. This kind of extensive benchmarking is beyond the scope of the current study.”

*- Line 88-89: Please add more references here, e.g. to the work by Sarkar or the subsequent discussion by the Weiss group.*

Thank you very much indeed! We have provided more references in lines 94-95 in the revised manuscript.

*- Line 137: The term "SBX crossover" has not been introduced.*

Thank you, the abbreviation is introduced in line 81 in the revised manuscript.

*- Figure 2: The resolution of the provided figure is too low to read the text.*

Thank you for pointing out this important oversight! We believe that this was an image conversion issue on the PlosOne website, we will make sure to double check the resolution after the new submission is completed. Please, note, that we cannot affect the resolution of figures in the pdf created by Plos submission system, however high resolution figures should be available from the submission.

*- Figure 2: Several things mentioned in the caption to figure 2 (e.g. mRNA expression), and adapting the patient-1 model for patient 2) have not been mentioned yet. The text would benefit from an introduction of these important concepts in the Introduction section.*

Thank you for pointing out this lack of clarity in our original submission. The revised Introduction section was expanded as mentioned above. New text in the lines 122-133 now reads:

“Finally, we have verified the algorithm against the experimental optical mapping AP recordings from the human heart. Since we could not measure ionic channel conductivities directly, we used the following assumption instead (Fig. 1B): these conductivities should be proportional to corresponding proteins mRNA level of expression as measured by Cap Analysis of Gene Expression (CAGE) [21] or RNA-seq. Thus, given that GA output model parameters represent actual ionic channels conductivities, the model rescaled in correspondence with mRNA expression profile differences between two patients, would reproduce AP restitution properties of both patients. Moreover, we have to note that this approach (i.e. combining GA with transcription profile) could be regarded as another technique of model personalization. As we show below, GA signal-to-noise ratio (SNR) requirements are rather strict and hard to accomplish in a clinical setting, while mRNA expression profile is possible to measure from tissue biopsy.”

*- Line 149: "coordinate descent" this term has not been introduced. Please rewrite to do so, or avoid using this term here instead.*

Thank you, we now provide a reference to the review paper, which describes coordinate descent algorithms on line 486 of the manuscript.

*- Line 160: "SD dynamics" this term has not been explained at this point in the text.*

Thank you, the “RMSE dependence on generation number” is used throughout the new version of the manuscript instead of the “SD dynamics”.

*- Line 166: "As mentioned above", the methods section is no longer above this sentence.*

Thank you, the methods section was shifted to the beginning part of the article.

*- Line 168: "Although the best organism is ... to the steady state.". This means that running the simulations has now become part of your mutation operator. This should be explained more clearly in the methods.*

Thank you for pointing out this lack of clarity in our original manuscript! We have now provided a more detailed explanation of elitism strategy in the Methods section. Please, see lines 232-238 of the text:

“More precisely: elite organisms do participate in mutation and crossover as usual, but an “unspoiled” copy is saved to replace the worst organisms in every generation. However, final state of elite organisms on generation N are still reused as initial state on generation N+1, thus slow variables get closer to steady state, while AP waveform and RMSE changes correspondingly (see “save state variables” above). We have found that using a high number of elite organisms (about 6-10% of the whole population) is optimal for our GA modification.”

*- Lines 200-201: Please rewrite this. 6-10% seems a fair estimate, but it is not demonstrated all that clearly from the graph (for example, what happens at ~7.5%?).*

Because of the random nature of the algorithm, any combination of algorithm parameters might accidentally result in fast or slow convergence for a particular run. We believe that slow convergence of algorithm at 7.5 % is a random fluctuation. We have revised lines 553-558 accordingly, while new S8 Fig demonstrates RMSE dependence on the number of elite organisms on generations 100, 150, 300 and 400 giving better sense of convergence to a reader.

“We observed slower algorithm convergence, when the number of elite organisms was below 4 % of the whole population. On the other hand, the increase of the proportion of elite organisms above 10 % typically resulted in algorithm stagnation after generation 100. Due to random nature of the algorithm convergence, the convergence speed is susceptible to fluctuations, but optimal proportion of elite organisms could be estimated as 6-10% of the whole population from S8 Fig.”

*- Lines 289-291: "The membrane potential differences are mostly confined to depolarization phase and are probably due to photon scattering". Please explain this in more detail, preferably with evidence to back it up.*

Thank you, more detailed explanation is added to lines 629-639, while (dV/dt)max values are provided to explain more clearly differences in depolarization. New revised text reads:

“The deviations between input data and the output model are listed in Table 1. While RMSE is close to the noise level, and APD80 error did not exceed 14 ms, the difference in the depolarization phase is very pronounced: (dV/dt)_MAX_ was approximately 20 V/s for input data, while for the output model it ranged from 55 to 80 V/s. This effect might be due to photon scattering in optical mapping recordings [10, 33]. Photons emitted by fluorescent dye undergo multiple scattering events and thus the recorded signal from a given pixel is actually an averaged signal from thousands of myocytes. This effect is known to distort AP waveform during depolarization when differences of membrane potential across the tissue are significant due to propagation of wavefront of excitation. Thus, in order to reduce the effect of this experimental artifact on the model, initial depolarization phase (below -20 mV) was removed from compared AP prior to fitness function calculation (see also Methods section).”

*- Figure 4: How much of the observed differences in convergence are due to the type of distribution, and how much due to the distribution's parameters? What would happen with a narrower Cauchy distribution? Or a wider polynomial one?*

We hope that new S9 Fig comparing RMSE with different distribution parameters provides the answer to this question.

*- Figure 6b: A central idea in genetic algorithms is that cross-breeding good solutions will yield improved solutions. The red trace in Fig 6B suggests that this is not the case for this problem. Does this imply that the crossover step is not beneficial for this problem? If so, it seems this is more a particle based search method rather than a genetic algorithm per se? It would be good if the authors could experiment with leaving out the crossover, or trying different optimisers on this problem.*

Thank you for this excellent suggestion! We have ran a number of additional simulations without crossover.S7 Fig depicts the results, while corresponding discussion is provided in lines 545-551 of the revised text:

“The requirement of a high number of elite organisms indicates that interbreeding via crossover operator does not necessarily result in improved results. In order to test whether crossover operator is indeed essential to algorithm convergence we have ran 7 GA runs without the crossover. Indeed, as seen in S7 Fig., leaving out the crossover still results in decent convergence, however some output parameters are much less precise, in particular the error is 100±50% vs 19±23% for IKs, 93±54% vs 32±25% for SERCA, 200±140% vs 26±14% for RyR.”

*- Figure 6: The resolution of the attached figure is too low to make this out clearly.*

Thank you, like in the case of Fig. 2 we will double check the resolution after conversion on PlosOne website.

*- Figure 6: These results are very interesting, and show that a GA with a number of elite organisms can act both as a global and a local optimiser. It would be good to discuss this further in the text. At the same time, the very fast convergence of elite organisms to something very close to the true solution suggests that it may be more efficient to perform a two-stage optimisation. Please comment.*

Thank you for the suggestion, this problem is discussed in lines 537-544 and 784-797 of the revised manuscript.

“In other words, large number of elite organisms result in two-stage optimization: initially the whole parametric space is explored, but eventually a number of elite organisms converge to the same minimum attracting the whole population to its vicinity and resulting in effective local optimization. Although PCA plots did not account for intracellular concentrations (see Methods section) it could be seen from comparison of S2 Fig (Cauchy mutation) and Fig 5 (6.6 % elite) that actually convergence to global parametric minimum (MCE and SDist reduction) is simultaneous with concentration changes to some vicinity of input model steady state value.”

“Albeit premature convergence to local minimum of RMSE hinders the solution of the optimization problem, it is preferable to exploit the vicinity of previously visited points once global minimum is localized. This is accomplished in this study by a large number of elite organisms. When elite organisms fall in some vicinity of RMSE minimum two scenarios are possible. If given solution is far from steady state they are going to be either discarded because of major variation of AP waveform in few generations (since these organisms are not susceptible to modification by mutation). If slow variables are close to steady state, elite organisms start to attract the population to the same vicinity. In the latter case, the whole population is going to exploit the vicinity of the optimization problem solution, thus working as a local optimizer. As seen from the comparison between S2 Fig. C-D and Fig. 5B-C intracellular concentrations and parametric convergence share similar dynamics when ratio of elite organisms is high. On the other hand, it might be more effective to use either classic gradient-based methods or Covariance Matrix Adaptation Evolution Strategy [39] once global minimum is localized (i.e. when a number of elite organisms converged to the same vicinity).”

*- Figure 7: It is not clear from the caption how this comparison was set up. Did you re-implement the method by Bot et al. and apply it to your data?*

Thank you, we have provided a more detailed description in the lines 561-567 of the revised text:

“We have re-implemented the original algorithm by Bot et.al. using Sastry toolbox [32] with the following modifications to it. Firstly, each organism was paced for 50 stimulations at every PCL, i.e. quasi-steady-state was used because it was computationally very expensive to reach actual steady state. Secondly, the normalized AP waveform at 7 PCLs was used as input data. Finally, the least squares technique was used to renormalize input data prior to fitness function evaluation as described in the Methods section.”

*- Line 391: "Computer simulations". Please provide more detail here. Why were 1D simulations preferred over single cell simulations, and why 1D instead of 2 or 3D? What integration method was used (and with what time step or tolerances?). How was this implemented? How many cells were stimulated, and from which cell or cells was an AP recorded?*

Thank you for this question. We provided detailed explanations on lines 200-206 of the revised text:

“1D model simulations, while being less computationally expensive than 2D or 3D models, correspond to a plane wave propagating in a 3D tissue at a significant distance from the pacing electrode. Moreover, given that in a wide range of conductivities (S1B Fig) exact AP waveform was essentially independent from gap junctions conductivity, we can conclude that in case of a minor 2D or 3D wavefront curvature, additional perturbations by diffusion operator would not affect AP waveform as well.”

*- Line 397-399: Please explain this shifting in far greater detail. Which of the two signals was shifted and did this happen once or at every iteration? (In the latter case, how did you stop this method from introducing identifiability problems or local minima?)*

Thank you, a more detailed explanation is provided on lines 178-188. One particular trick that we used is assigning large RMSE values to subthreshold depolarizations. AP waveform rescaling was actually performed for every organism. Please also find a brief discussion on the identifiability problem in lines 102-121 of the new manuscript.

“The algorithm is optimized for optical mapping recordings, where absolute transmembrane potential (TP) values are not known, and thus input data (both synthetic and experimental) was normalized by the algorithm. The least-squares technique is used to find scaling and shift coefficients of AP waveform. For every organism, before fitness function evaluation, input AP was shifted along the time axis to superimpose half-maximum depolarization of compared waveforms, after that experimental AP is rescaled and shifted to minimize the deviation between the experiment and the model: , where	and	coefficients are determined by the least-squares technique. In order to discard subthreshold depolarizations some large error value was assigned to low-amplitude APs (see below “Fitness function” subsection).”

“To the best of our knowledge, this is a first study providing a technique suitable for optical mapping recordings, where only normalized AP waveform is known, but not the exact transmembrane potential values. Arbitrary rescaling and shift of input AP waveform introduces a new dimension to parameters identifiability problem mentioned above. A possible approach to solve this problem is to utilize so-called restitution property, which is AP dependence on heart rate or pacing cycle length (PCL). For example, the sodium current reduction would result not only in the reduction of the amplitude of AP, but also in change of the steady state intracellular ionic concentrations and consequently, in the restitution curve change. Previously, several publications [2,5] utilized restitution property for GA-based cardiac model optimization. For example, Syed et. al. [2] have used atrial AP waveform at several PCLs as input for GA and paced every organism for 10 seconds before fitness function evaluation.”

*- Line 407: This is not a standard deviation but a root-mean-squared error.*

Thank you, SD was changed to RMSE in text and figures.

*- Line 407: The choice of the symbol t^i_max for the number of samples (which is not a time) is confusing.*

Thank you, the symbol was changed to *N.*

*- Lines 419-420: "we have found odd number of stimulations helpful to exclude possible 1:1 alternans in the output model." This needs a lot more explanation.*

Thank you, we have provided additional explanations on lines 223-226:

“in the case of alternating APs, the waveform (and, consequently, RMSE as well) was different every other generation. Thus, if alternating AP have a low RMSE value on generation N, the RMSE is going to increase on generation N+1.”

*- Line 430-431: "This is especially important in terms of intracellular ionic concentrations" Why?*

As already mentioned above in this reply to reviewers we have substantially revised the section of the article describing the Cauchy mutation.

*- Line 430: Local minima are a problem _for_ optimisation methods, not _of_ them (they are a property of the score function being optimised).*

Thank you, the error was fixed, however these lines were removed in the revised manuscript.

*- Lines 434-438: Please add more detail. What is a "half-width of the half-maximum of the distribution", and why is 0.18 times the original value a good choice?*

The explanation is provided in line 246. “(*i.e.* PDF is half the maximum value, when $x-x_{0}=\gamma$ )” We have also provided additional S9_Fig demonstrating convergence dependence on γ value.

*- Line 441: "We have found that it has adverse effects on algorithm convergence". Please explain where/if this is shown.*

Thank you a reference to corresponding Results section is provided on line 259 of the revised manuscript: “See also Fig. 3 and the corresponding Results section.”

*- Lines 440-444: Please list the parameters that were varied.*

Thank you. The list of parameters was provided in lines 237-240 of the revised text:

“Thus, when input data included 4 pacing frequencies the full list of parameters was: gNa, gKr, gK1, gKs, PCaL, gto, gNaK, gNCX, gpCa, Jrel, Jup, CMDN, CaMKII, [Na^+^]_300 ms_, [Na^+^]_500 ms_, [Na^+^]_1000 ms_, [Na^+^]_2000 ms_, [Ca^2+^]_300 ms_, [Ca^2+^]_500 ms_, [Ca^2+^]_1000 ms_, [Ca^2+^]_2000 ms_.”

*- Lines 441-443: Please explain how applying a mutation to each parameter seperatly differs from your approach. Is the mutation in a parameter i now correlated to the mutation in parameter j somehow?*

Additional explanations were provided on lines 257-259:

“For example, in the case of the two-parameter problem: if ($\frac{\sqrt{2}}{2}$,$\frac{\sqrt{2}}{2}$) unit vector was chosen, then both parameters are going to be increased by the same amount after mutation. See also Fig. 3 and the corresponding Results section.”

- Line 453: 1000s? Or 1000*PCL ms?

1000 seconds. We have changed “s” to “seconds” in the text.

- Lines 456: The Andrews plots are cool, but don't offer much insight into which parameters vary. Did you try simpler representations, e.g. radar plots, instead?

Thank you for this suggestion. Radar plots are provided in the revised Fig. 6.

- Line 466 m x n * p. Please add parentheses and use x or . instead of *.

Thank you, we have changed the formula to *mxnp* on the line 289.

- Lines 524-525 "Libaries in silico processing performed in Morai system.". Please rewrite this.
- Line 529: "python scripts: level1 and level2". What do these scripts do?

Thank you: the CAGE data processing was modified on lines 371-372 and 377-380:

“In silico processing of sequenced CAGE tags was performed by using Moirai system [24].”

“The first script generates CTSS (CAGE tag starting sites) files, where 5' end of the mapped CAGE reads are counted at a single base pair resolution. The second script performs signal clustering on CTSS files with a minimum 10 TPM (tags per million) in at least one sample and minimum distance between clusters of 20 base pairs.”

- Line 541: "(100 s)", 100s or a 100 beats?

We have changed it to 100 seconds, see line 401.

- Line 558: "below". This is no longer below.

Thank you, the Methods section was shifted to initial part of the article.

We would like to thank Reviewer #1 for most thorough and helpful review, which significantly improved our manuscript!

***Reviewer #2:*** 1. Is the manuscript technically sound?

I believe it is. There were multiple moments reading through the paper where I began wondering if the authors had considered something I considered an issue, and then found that they had as I continued reading. I consider this a good sign that the application of the genetic algorithm technique to the problem of personalised cardiac model generation here has been considered in a good amount of depth. The methodology also proves quite successful.

Although there are some points below where I comment on potential issues with the methodology, I do not feel that they compromise the core material of the manuscript (application of a modified GA to achieve action potential model personalisation). The authors should, however, clarify what is going on with the question raised on Line 123.

I cannot comment on the experimental procedures (obtaining imaged voltage data or genetic expression levels).

Thank you for positive assessment of our work. We have thoroughly revised our manuscript to address your concerns and concerns of other reviewers.

-----------------------------------

2. Has the statistical analysis been performed appropriately and rigorously?

I believe so, but it would improve the paper if the results were also presented in a statistical way (i.e. in text, comment on spread of performance across runs, instead of using the worst run as the discussed figure).

Thank you! Mean and SD values are now provided in the revised manuscript.

-----------------------------------

3. Have the authors made all data underlying the findings in their manuscript fully available?

I missed where in the paper this was discussed, but am trusting the authors when they state on the online submission that data has been made available. If it is not yet mentioned in the paper, I suggest a very short (1-2 sentence) new section at the end of Methods that points readers to where data can be obtained.

Yes, we have uploaded the data to datadryad.org. The data is going to be available upon article publication with the following DOI <https://doi.org/10.5061/dryad.stqjq2c09> . Meanwhile the data is available to reviewers and editors via the following link <https://datadryad.org/stash/share/LZQuhX_jA7dq003OcNniS7B8ngFfIy3J6hEY4644x5o> .

“Since the manuscript involves human subjects research, the data disclosure is sensitive. We will share the data with anyone upon the request to one of the corresponding authors.”

-----------------------------------

4. Is the manuscript presented in an intelligible fashion and written in standard English?

I feel the work is rather clumsily written. There are many grammatical errors, but those are a separate issue. What I consider more important is that as a reader, I was given very little idea about how the paper sits within the literature. For example, a list of references of other works using the genetic algorithm in the cardiac context is provided, but then only one is further discussed (as the method there serves as the basis for the authors' modified method). Without going through all of those papers, a reader will have no idea whether those works used experimental/clinical data, or simply demonstrated an ability to recover chosen parameter values in the action potential model (i.e. fitting synthetic data). Flicking through the referenced papers, they do seem to work with experimental data, and so this is not a point of novelty.

Thank you for this important critique! We have extended introduction section and hope that new revision gives a better sense of novelty and reviews previous literature in the field. These are the main points of our motivation:

Firstly, to the best of our knowledge this is a first study discussing GA application to optical mapping recordings of human action potentials, where only a normalized AP waveform is available, but not the exact voltage values. Secondly, in the previous reports which relied on experimental data to unambiguously determine computer model parameters, only single-cell voltage-clamp recordings were utilized. That limits the scope of the GA method application. Most importantly, since it is almost impossible to measure all ionic channels conductivities none of the previous works verified output parameters of GA as applied to experimental data. Please, find new text in the lines 94-133 of the manuscript:

“Another limitation of optimization algorithms as applied to electrophysiological models is the absence of a unique solution. As was noted previously [8–10] same AP waveforms could be reproduced by computer models with different sets of parameters, in other words, model parameters are unidentifiable from the AP. Also, techniques combining stochastic pacing and complicated voltage-clamp protocols have been recently proposed to overcome the problem [4,8]. However, these techniques are limited to single-cell voltage-clamp recordings, which are not feasible in clinical electrophysiology or whole heart and cardiac tissue measurements. The aim of the current study was to develop a technique that would allow one to find a unique solution using optical mapping, microelectrode, or monophasic AP recordings from cardiac tissue or whole heart. To the best of our knowledge, this is a first study providing a technique suitable for optical mapping recordings, where only normalized AP waveform is known, but not the exact transmembrane potential values. Arbitrary rescaling and shift of input AP waveform introduces a new dimension to parameters identifiability problem mentioned above. A possible approach to solve this problem is to utilize so-called restitution property, which is AP dependence on heart rate or pacing cycle length (PCL). For example, the sodium current reduction would result not only in the reduction of the amplitude of AP, but also in change of the steady state intracellular ionic concentrations and consequently, in the restitution curve change. Previously, several publications [2,5] utilized restitution property for GA-based cardiac model optimization. For example, Syed et. al. [2] have used atrial AP waveform at several PCLs as input for GA and paced every organism for 10 seconds before fitness function evaluation. As we demonstrate below in the Final Algorithm subsection of the Results section, this approach results in a poor parameters convergence. We have identified two reasons behind this fact: firstly, intracellular concentrations require much more than 10 seconds to converge to a steady state; secondly, a model with the particular set of parameters may converge to different steady states depending on the initial conditions. In order to address these issues we implemented a modification of GA allowing one to perform parameters optimization and steady state search in the slow variables space simultaneously. After each short simulation variables are saved, modified by genetic operators and reused as initial states for a new generation.

Finally, we have verified the algorithm against the experimental optical mapping AP recordings from the human heart. Since we could not measure ionic channel conductivities directly, we used the following assumption instead (Fig. 1B): these conductivities should be proportional to corresponding proteins mRNA level of expression as measured by Cap Analysis of Gene Expression (CAGE) [21] or RNA-seq. Thus, given that GA output model parameters represent actual ionic channels conductivities, the model rescaled in correspondence with mRNA expression profile differences between two patients, would reproduce AP restitution properties of both patients. Moreover, we have to note that this approach (i.e. combining GA with transcription profile) could be regarded as another technique of model personalization. As we show below, GA signal-to-noise ratio (SNR) requirements are rather strict and hard to accomplish in a clinical setting, while mRNA expression profile is possible to measure from tissue biopsy.”

Instead, a potential point of novelty is the consideration of steady state of the action potential model. The methodology in this paper has been specifically adjusted to recover the steady state as it goes, something which I suspect is novel. So then the questions are, is this indeed novel? If so, the authors should highlight it in the introduction, by pointing out other genetic algorithm applications in cardiac electrophysiology have not considered it (or considered it simply by incurring great computational cost). Additionally, if steady state is so important, the authors should highlight it. Figure 1 shows the effects of choosing different initial conditions, but that is a different issue - if anything, re-using the final state of an organism as the starting point for its offspring seems like it exacerbates this issue (more comments on this below).

Thank you, and yes – this is novel. We have modified the figure (Fig 2 in the revision) and corresponding text according to your recommendation. See text in lines 456-462 of the revised text:

“Why would this technique eventually result in concentrations converging to a steady state? The rationale is the following: if given parameters vector results in an acceptable AP waveform (i.e. close to the input AP), but far from steady state, then this solution is going to be discarded eventually, since waveform is going to change after few beats. For example, if AP of particular organism on generation N minimizes RMSE, but corresponds to one of the dotted lines in Fig. 2B, then RMSE is going to be large for this particular organism on generation N+1 (one of the dashed lines). “

Modifications are made to the genetic algorithm to improve its performance, but again it is not at all clear whether these are only novel in the field of cardiac electrophysiology, or novel ideas of the authors in the GA context. For example, using Cauchy distribution jumps is something I've seen in other optimisation (not GA) contexts (e.g. "Levy-flight" jumps in bio-inspired optimisation algorithms), and I assume has also been used in the GA context already. The authors should check up on this if they haven't, and provide an appropriate reference. Similarly, random vector jumps might indeed not be the norm in GA, but I'm sure they must've been used (many optimisation algorithms, including many that use multiple search agents as GA does, will be doing random multivariate Gaussian jumps, for example). Additionally, if these two modifications were so necessary to get good results here, one wonders how some of the other published GA papers in cardiac electrophysiology got away with not using them (or if they did use them, this should definitely be mentioned and emphasis on those aspects of the paper reduced!).

Thank you for this important point, which we missed! A brief discussion of the subject is now provided in the Discussion section of the revised manuscript as follows:

“It is worth noting that all these modifications to GA essentially make the algorithm similar to particle-based algorithms. For example, multivariate jumps are used by Particle Swarm [38], Covariance Matrix Adaptation Evolution Strategy [39] and Cuckoo Flight [40] algorithms. The last one uses “pathological” distribution similar to Cauchy mutation for Levy-flight walk. Although it would be very interesting to compare these algorithms performance with GA modification proposed in this study, some custom modifications to the aforementioned algorithms are still required: as was noted above same AP waveform could be reproduced by different parameter sets [8]. In order to narrow down the output parameters range, not steady state solutions should be somehow automatically discarded by the algorithm. This kind of extensive benchmarking is beyond the scope of the current study.”

Instead, one major novelty of this work seems to be the success of a GA-fitted model to be used across different members of a population, simply by measuring differences in their relative gene expression levels. Although I don't have a great sense for how easy/hard this is to actually do clinically and so cannot comment too much on the impact level of this finding, this was certainly a unique (and interesting!) aspect of the paper... and one that isn't even mentioned in the introduction! Hence my comment about clumsy writing. As I state many times in this review, I feel this part of the work needs to be highlighted much more.

Thank you very much for this recommendation! We agree completely! The reason we have not emphasized this point earlier was that previous data was not extensive enough to provide well-grounded discussion of the subject. Now during revisions, we were able to collect additional data in address reviewers’ concerns. Please see new Figs 11-12 and corresponding sections of the text. Introduction, Methods, Results and Discussion were all extensively revised to cover this subject.

I also re-iterate that although the English is mostly understandable, there are many grammatical errors and that the manuscript could be improved significantly in this regard. I have highlighted some of the most glaring issues below.

Thank you very much for your numerous helpful recommendations that allowed us to improve the paper significantly, both scientifically and stylistically/grammatically.

-----------------------------------

Specific Issues:

*Abstract: It feels a bit number-heavy, and the numbers quoted are not average (with standard deviation) error, but maximum error, which I don't think is typical. It also talks about things like "13mV accuracy" which doesn't make sense to me. Is that a maximum deviance of 13mV at any point along the action potential? A root mean square error of the voltage along the whole action potential? This is not clear. In general, I personally think the "story" of the paper could be much better told than just listing error numbers.*

Thank you, we have modified the abstract accordingly.

*Line 50: There's no guarantee the parameters found by GA (with the benefit of multiple pacing frequencies) will be unique, but indeed use of multiple pacing frequencies helps a lot. If this wasn't explicitly considered, I recommend softening this language (similar to how it's expressed on line 97).*

Thank you, we have modified the text as you recommend. New lines 50-51 read:

“In order to find the set of model parameters we use steady-state action potential waveform dependence on heart rate, known as restitution property.”

*Line 98: Again, the intro doesn't mention use of CAGE at all. This seems like a huge mistake!*

Thank you, we have provided the discussion of the subject in the lines 122-133 of the revised text:

“Finally, we have verified the algorithm against the experimental optical mapping AP recordings from the human heart. Since we could not measure ionic channel conductivities directly, we used the following assumption instead (Fig. 1B): these conductivities should be proportional to corresponding proteins mRNA level of expression as measured by Cap Analysis of Gene Expression (CAGE) [21] or RNA-seq. Thus, given that GA output model parameters represent actual ionic channels conductivities, the model rescaled in correspondence with mRNA expression profile differences between two patients, would reproduce AP restitution properties of both patients. Moreover, we have to note that this approach (i.e. combining GA with transcription profile) could be regarded as another technique of model personalization. As we show below, GA signal-to-noise ratio (SNR) requirements are rather strict and hard to accomplish in a clinical setting, while mRNA expression profile is possible to measure from tissue biopsy.”

*Line 110: It's nice to point out the issue with a sensitivity to initial condition in cardiac AP models. I believe this is an issue that has been commented in the literature, although I cannot think of a reference off the top of my head. One would be nice here (see also comment on Line 120, though).*

Thank you, although this issue is rarely considered, we have provided a reference to an important Rudy lab’s paper addressing the subject. Please, see lines 445-446 of the revised text:

“We have to note here that, while sensitivity of cardiac models to initial conditions is rarely considered, is was discussed previously [30].”

*Line 114: "Saving organisms state vectors" is not a good figure title, in my opinion. Most of the figure is discussing the behaviour of the action potential model. Speaking of, I feel the caption should mention the model used (O'Hara-Rudy), given the text hasn't yet, and the reader might get curious. I know I did.*

Thank you for noticing this glaring omission on our part! The figure caption was modified accordingly.

*Line 120: My issue here is, I don't see how the methodology introduced here actually helps with the issue just discussed, of different initial conditions giving different steady state behaviour. If anything, using the final state of an organism as the initial condition of its offspring seems like it will only cause the method to hone in on a certain behaviour, and fail to explore the range of possibilities represented by different initial conditions. I do not consider this a major failing of the work, as I do not think there are many works at all in cardiac electrophysiology that even consider, let alone deal with this issue. Instead, what I think should happen is that the issue is given less focus in the manuscript (if even brought up), and instead, Fig. 1A-C should highlight the importance of steady state (for example, by plotting an action potential at steady state, and one during the transient phase). Assuming steady state even is important, but one is lead to believe it is in this paper, given the focus on achieving it whilst also avoiding excessive computational cost. This change would make Fig. 1 contribute a lot more to the story of the paper.*

Thank you! Former Fig. 1 (Fig 2 in the revisions) was modified accordingly. New figure depicts initial and intermediate AP waveform in addition to the steady state. Revised text was also provided in the lines 449-456. Briefly, the offspring initial state is also inherited from the final state of the parent, in between the fitness function evaluations (model runs) the concentrations are modified by mutation and crossover operators, prohibiting certain behavior of the model. Revised text now reads:

“However, given that the set of parameters minimizing RMSE is itself a function of intracellular concentrations, this approach results in an optimizer solving essentially a new problem every generation. Moreover, as shown above (Fig. 2 B-D) “fixed” initial state for the organisms should result in a “fixed” steady state that might be different from input data. The solution that we propose in this study is to perform a simultaneous search in the parametric and slow variables space, i.e. the initial values of slow variables were added to parameters vector making them susceptible to mutation and crossover operators.”

*Line 123: It was not originally clear to me how allowing intracellular ion concentrations to vary would speed up convergence. I now see that this is about convergence to steady state over the course of the genetic algorithm. Perhaps this could be reworded somehow to be more clear. Furthermore, I don't understand how [Na+]_i and [Ca2+]_nsr can be parameters chosen by the GA, given that they are variables in the model. Do the authors mean the *initial* value is allowed to vary within the GA? Or am I missing something? This needs to be made clear in the paper.*

Thank you, we hope new lines 456-469 discuss the issue much more clearly. Revised text reads:

“Why would this technique eventually result in concentrations converging to a steady state? The rationale is the following: if given parameters vector results in an acceptable AP waveform (i.e. close to the input AP), but far from steady state, then this solution is going to be discarded eventually, since waveform is going to change after few beats. For example, if AP of particular organism on generation N minimizes RMSE, but corresponds to one of the dotted lines in Fig. 2B, then RMSE is going to be large for this particular organism on generation N+1 (one of the dashed lines).

In particular, we suggest including the initial intracellular sodium ([Na+]i) and calcium sarcoplasmic reticulum load ([Ca2+]NSR) at every PCL as model parameters. The [Ca2+]NSR concentration was chosen, because most of the calcium is stored within sarcoplasmic reticulum when the cell is at resting potential. We did not include potassium concentrations in the optimized parameters vector, since 10 mM [K+]i concentration changes results only in approximately 2% Nernst potential change, and thus a major concentration changes have a minor immediate effect on AP waveform.”

*Line 125: "1.5mM precision" sounds not great for an intracellular ion concentration (well, maybe [K+]_i!). This seems to be the worst possible value, which is not a great way to report your error, in my opinion. Mean and standard deviation makes a lot more sense (and will look better!)*

Thank you, we have modified maximum values to mean and standard deviation throughout the text.

*Line 132: SD is not defined yet as far as I saw.*

Thank you, the SD was changed to RMSE in new version of the text, while the methods section moved to initial part of the article.

*Line 141: Patient 1 and patient 2 seem to be switched here (or in the figure). This was also an issue when the CAGE results were discussed later.*

Thank you for pointing out this error! The Patient 1\2 error was fixed in Fig.1 caption as well as 644-646 of the text.

*Line 154: Again, these error values seem to be worst-case.*

Thank you, the issue was fixed. Please see lines 506-508 of the manuscript:

“For example, the error of model parameters was 14±24% vs 17±75% for IKs, 11±25% vs 45 ±23% for Ito, 6±6% vs 13 ±15% for ICaL in GA runs with Cauchy and polynomial mutation correspondingly.”

*Line 178: "Proper value" is mentioned here. Is that the value for synthetic data? I think this needs to be clearer.*

Thank you, the issue was fixed. See lines 504-506 of the text.

“Consequently, when synthetic AP was used as input data, we observed more robust algorithm convergence in case of Cauchy mutation (Fig. 6B).”

*Line 181: It's interesting that the best model organism not changing (implying stagnation of the algorithm) is here pointed out as a good thing. Can't argue with the results, though!*

Actually, the stagnation of the algorithm itself was not pointed out as a good thing. Instead, we were drawing attention of the reader to the fact that in this example, it is clear that mutation did not spoil the convergence to the steady state. However, we have ran additional tests and noticed that although Cauchy mutation in general resulted in an output state closer to steady-state, the difference was not statistically significant at the higher numbers of generations. Therefore, Cauchy mutation section was modified substantially in the new version.

*Line 215: The point here about "fine tuning" needs to be expressed better. I think I understand it now, but it took me a few reads. The idea is that with a small cluster, and offspring generated from the current population of organisms, the algorithm will only search locally and thus waste less time, right?*

Yes, this is exactly what we meant; we have modified the text to express it more clearly. See lines lines 524-528 and 537-540 of the Results section, and lines 784-797 of the Discussion section:

“While wide exploration of parametric space is required at the initial stage of algorithm convergence, it is more effective to exploit the global minimum once it was localized (see [31] for discussion on exploration and exploitation). This could be achieved by a large number of elite organisms passing their parameters via crossover operator to siblings after clustering around the same minimum.”

“In other words, large number of elite organisms result in two-stage optimization: initially the whole parametric space is explored, but eventually a number of elite organisms converge to the same minimum attracting the whole population to its vicinity and resulting in effective local optimization.”

“Albeit premature convergence to local minimum of RMSE hinders the solution of the optimization problem, it is preferable to exploit the vicinity of previously visited points once global minimum is localized. This is accomplished in this study by a large number of elite organisms. When elite organisms fall in some vicinity of RMSE minimum two scenarios are possible. If given solution is far from steady state they are going to be either discarded because of major variation of AP waveform in few generations (since these organisms are not susceptible to modification by mutation). If slow variables are close to steady state, elite organisms start to attract the population to the same vicinity. In the latter case, the whole population is going to exploit the vicinity of the optimization problem solution, thus working as a local optimizer. As seen from the comparison between S2 Fig. C-D and Fig. 5B-C intracellular concentrations and parametric convergence share similar dynamics when ratio of elite organisms is high. On the other hand, it might be more effective to use either classic gradient-based methods or Covariance Matrix Adaptation Evolution Strategy [39] once global minimum is localized (i.e. when a number of elite organisms converged to the same vicinity).”

*Line 304: This is not a clear way to discuss error, as commented on previously.*

Thank you, the issue was fixed.

*Discussion: I guess it is up to the editor and journal as to whether this numbered list of highlight points is suitable format for a discussion. It's not my personal preference.*

We have modified the discussion, accordingly.

*Line 330/343/350: I'm not sure that the modifications to the GA are worth three separate discussion points. In particular, discussion points #3 and #4 should probably be combined and cut down a little, in my opinion.*

Thank you. We have removed some parts of the text from discussion, however, after considering reviewers questions, we have decided that some points on algorithm modification have to be discussed more clearly. Thus, the discussion was cut down and extended once again. Please see lines 758-815 of the text.

*Line 358: "algorithms" should be "organisms". There are many more grammatical issues, but this one is one that changes the meaning so I have commented on it specifically.*

This part of the text was removed according to previous critiques.

*Line 376: Tacking on the CAGE results at the end here with a casual mention is not really doing them justice. Those results seem to me like a much bigger deal than the authors make them sound!*

Thank you, as mentioned above, we have performed additional experiments and extended corresponding sections.

*Line 466: This notation was unclear to me. Combining two different multiplication symbols leaves me guessing which is which. If I guessed right, I think you could just say m x np?*

Thank you for the suggestion, we have fixed the text accordingly. Please, see line 289 of the new version of the manuscript.

We would like to thank Reviewer #2 for a thorough and friendly review, which served us a welcome guide during revisions. We hope you find it improved.
