# Supplementary material for: Genetic algorithm-based personalized models of human cardiac action potential
Source: PLoS One. 2020 May 11;15(5):e0231695. doi: 10.1371/journal.pone.0231695 (PMC7213718; doi:10.1371/journal.pone.0231695)
Supplement: Attachment — Submitted filename: Response to Reviewers.docx [file pone.0231695.s016.docx]

We are grateful to the editor and reviewers for the opportunity to revise and resubmit our manuscript. In order to expedite the review process we restated reviewers’ questions in green font and our provided our answers in blue font.

Please use the space provided to explain your answers to the questions above. You may also include additional comments for the author, including concerns about dual publication, research ethics, or publication ethics. (Please upload your review as an attachment if it exceeds 20,000 characters)

Reviewer #1: I'd like to congratulate the authors on a much improved manuscript and a very interesting study.

We thank the Reviewer very much for the positive assessment of the revised manuscript.

A few minor concerns remain, which I am confident the authors can address:

- The caption to figure 2 has not been updated. (B,C) should now be (C,D), (D,E) should become (E,F), and the new B panel should be described.

Thank you, we have fixed the error in figure caption.

- The first and last sentence of lines 218-228 appear almost contradictory, please rewrite.
Thank you, we believe that we have expressed the idea more clearly in the new revision, new text reads as follows:

“Computational cost of pacing every organism at every generation until reaching steady state during a GA run is prohibitively high. Thus, we saved each AP after a short simulation… As a result, each organism approaches closer to steady state variables with every generation.”

- The statement on line 634-636 needs clarification

Thank you, we have included a sentence explaining the statement:

“One possible explanation of this heterogeneity is uneven perfusion of the sample resulting in a mild ischemia that shortens AP duration because of activation of the ATP-dependent potassium channels I_K,ATP_.”

Finally, time permitting, the authors might consider adding a comparison of GA-obtained parameters with measured RNA levels (e.g. show if/how they correlate).
But as I didn't mention this in the initial review I'm happy to let this go!

Indeed, we have considered the correlation between output parameters and mRNA levels. However, given the limited amount of experimental data, we concluded that correlation is not too meaningful. As we mentioned several times throughout the text, we believe that at least in some cases experimental tissue was exposed to ischemic conditions. We also demonstrate in the article that Patients 9 and 11 models performance are not perfect: the former underestimated the sodium current, while the latter overestimated the APD. Moreover, as we demonstrate in Fig. 6, the precision of some parameters, such as low-amplitude ionic currents, was relatively imprecise. We thus concluded that, given the limited amount of data, model rescaling is the best way to verify the algorithm precision. For example, low-amplitude currents would spoil the correlation, but are less likely to affect AP waveform after the parameters rescaling. However, we will address this important issue in the future studies.

Reviewer #2: Firstly I thank the authors for their very thorough response to my comments, and I believe that they have addressed the points I raised very well. The introduction reads a lot better now, in particular, and the discussion is also much improved. The improvements made to figures really help, also!

However, the paper has now also added a lot more results and I feel this has introduced some new issues into the work. I actually wonder if there was a misunderstanding of my (and the other reviewer's) comments - from what I see both of us asked for more emphasis to be placed on the CAGE results, but this meant (certainly in my case anyway) to give these results a proper discussion, mention them in the introduction, and so on. Instead, the authors have now included many more results further testing out the idea of using CAGE and also RNA-seq data to attempt to generate personalised models. This is great! But still actually feels underemphasised in terms of the text (things like discussion of the implications is a bit lacking). I would like to iterate how impressive this result is! I feel the impact of the paper could be greatly improved if more of a focus was given to those results. That said, PLoS One is about publishing correct science without all the emphasis on impact, and so I am not considering this a negative so much as a comment as to how I think the paper could potentially be further improved.

I have selected a recommendation of Minor Revisions. Although I have perhaps a lot of comments below, I would like to express the fact that I think the paper is much improved and with some cleaning up absolutely fit for publication.

We thank the reviewer for this positive evaluation of the new revision and new valuable comments and recommendations. We have carefully considered the reviewer’s recommendations and modified the manuscript accordingly.

I have some general comments, and then specific comments that I have sorted into important, and less important.

General Comments

----------------------------------------------------------------------------------------------------

* I think the referencing of the supplementary figures is out of order. Not a huge deal, but suboptimal. Additionally, the style of referencing figures is not consistent throughout the paper. This would need to be fixed up in review or proof stage.

Thank you, we have fixed the order and formatting of the references.

* The paper was originally written to have the methods at the back, and this was now changed for this version. However, some of the text in the Results feels like it's there because it's a holdover from the previous revision (or reworked/improved text from the previous version). This has resulted in text in the Results that feels more like justification for choices made in Methods and I think it would improve the paper to move it back into Methods, now that Methods comes before Results.

Thank you, we have moved some portions of the text to methods, in particular the description of the slow variables mutations was shifted to the “Methods” section as recommended by the reviewer. While we agree that on many occasions “Results” section repeats some of the information already mentioned in the “Methods” section, we have to note, that half of the figures is indeed the validation of the GA modifications. Although, mRNA-related study is probably much more impressive, we believe that these modifications were crucial to the successful model rescaling, and thus this validation was necessary. In our opinion, in most of the cases repeating some information from the “Methods” was necessary to spare the reader from flipping pages back and forth.

* The English is on the whole understandable and again I think the writing was improved, things I picked up on last time were given a much better explanation. That said, I still believe there is considerable room to improve the grammar, which would help in making the paper more readable, but also in selling the results and conclusions to the reader. I still consider it the journal's call as to what standard of grammar is acceptable.

For now, I have marked the criteria for "presented in an intelligible fashion and written in standard English" as a No. This is less because of the grammar and more because there are some specific issues (covered in my comments) that I think need to be fixed first. However, I do think the paper is understandable on the whole and I would mark it "Yes" if not for those specific issues.

* My other major comment relates to the arbitrarity of the results that are/are not displayed for the CAGE and RNA-seq model personalisation sections. Some of this might have been me missing something, but why is only Patient 2 compared to data, and not Patients 3-7? Was analysis run using one of the other patients as the baseline (as done for the RNA-seq section?). If so, transparency as to how that went would be good, and if not, this should be stated so that the reader isn't left wondering if Patient 1 was cherrypicked as the best.

Similarly, in the RNA-seq section, why are only patients 8, 9 and 11 used as baselines to create the other models? I appreciate that too many lines in Figure 11 would make it unreadable, but did the authors try using the other patients as baselines also? It would be good to have this at least commented on. Whether a sentence admitting that the three shown were the best three, or that "performance using the other patients as baselines was mixed, and not as good as patients 8 and 9", or whatever the exact case is here. If the analysis wasn't run for those, then say that, so that it doesn't just look like cherrypicking.

We thank the reviewer for pointing this omission on our part; we have revised the Methods section to avoid misinterpretation of the actual results, new text in the lines 411-413 and 421-427 reads:

“In the case of CAGE group of patients, functional data was not available for *Patients 3-7*, thus only *Patients 1 and 2* were used for algorithm verification.”

“Due to computational limitations, after preliminary analysis several patients available were excluded and not used as input to GA. In particular, *Patient 10* was excluded because of a very long depolarization time (see “Experimental data” subsection of the “Results” section for a brief discussion of optical mapping artefacts affecting depolarization phase). *Patients 12* and *13* were excluded because of the very short APD (below 300 ms, which indicated ischemia of the preparation). *Patients 12* and *14* were excluded due to low signal-to-noise ratio, while at high frequencies alternans was also observed for *Patient 14*.”

* Figure 11 is super low quality in the version I have here. Not sure if it was corrupted as part of the submission process, or if the figure just needs to be created in higher quality for upload. As it stands it's very hard to visually make out some of what it shows (for example, + and x are indistinguishable, and the lines of different colours are hard to tell apart, all can sometimes just appear grey due to image artifacts).

We are sorry, but actually, we believe the Figure 11 (Figure 10 in the new revision) resolution to be very high. Although we cannot control the conversion from TIFF to the PDF on the PLOS One site, we suggest downloading the high definition figure via the link available from the PDF above the figure. However, we are not sure if the link works for reviewers.

* Table 5 and surrounding discussion need work. See the relevant specific comments below.
Thank you we have modified the table and the text according to suggestion.

Specific Comments (Important)

-----------------------------------------------------------------------------------------------------

Line 39: This final sentence could be written in a far more impactful way. It also still mentions two patients, even though far more were used in the current results.

We have modified the abstract according to suggestion, new version reads:

“We have demonstrated that mRNA-based models predict the AP waveform restitution with high precision. The latter also provides a novel technique of model personalization that makes it possible to map gene expression profile to cardiac function.”

Line 65: It is simply not true that single-cell models "remain non-personalized". Line 71 is reviewing other papers who have used GA to recapture an experimental AP.

Thank you for pointing out this inaccuracy, we have removed the sentence from the text.

Line 91: Weird phrasing. The goal of the study is not to "develop a GA implementation suitable for cardiac models", because those already exist and are referenced. I just think this wording is a little strong and also not very specific to the very real benefits your GA approach brings.

Thank you we have changed the text in the lines 89-90:

“One of the goals of the current study was to develop robust GA implementation making it possible to find the set of cardiac electrophysiology model parameters without premature convergence to sub-optimal solution.”

Line 171: (B,C) in caption should be (C,D) I think

Line 172: (D,E) in caption should be (E,F) I think

Thank you! The error in the figure caption was corrected.

Line 175: This mentions green-tinted boxes which were in Fig. 1A, not 2A. Fix numbering.
Thank you, the numbering was fixed.

Line 221: Use something like "infeasible" instead of "impossible". Something is not "computationally impossible" just because it takes a long time.

The text was fixed according to suggestion. Please, see line 216 in the new revision.

Line 425: I was first left wondering why there were essentially two groups, patients 1-7 and then 8-14. I eventually realised that this was because one group was using CAGE, the other RNA-seq. This could really have been expressed much more cleanly at some point around here.

Thank you, the clarification was added to the methods section, lines 402-404:

“Genome-wide transcription profile was measured *via* CAGE (for *Patients 1-7*) or RNA-Seq (for *Patients 8-14*) techniques as described above.”

Line 445: I appreciate adding in a reference discussing the issue of sensitivity to initial conditions as I suggested. However the sentence that does so reads very awkwardly and reads very much like one of those "here because reviewer wanted it" sentences. Why not delete this sentence, and instead just add this reference on to the end of the sentence on line 438-439 where you're actually naturally bringing up this point about initial conditions in the first place? Something like:
"Moreover, given different initial states, intracellular concentrations can converge to different steady state values [30], although this issue is very often neglected in cardiac electrophysiology studies."

Thank you, we have changed the text according to your recommendation.

Line 462: From what I saw in the figure, moving from dot to dashed line is not "one generation" as the text implies here.

Actually, since 9 stimuli were used for every generation, moving from dotted to dashed line is a single generation indeed. We have added a brief explanation that we hope will clarify this issue (line 460):

“For example, if AP of particular organism on generation N minimizes RMSE, but corresponds to one of the dotted lines in Fig. 2B, then RMSE is going to be large for this particular organism on generation N+1 (***i.e.* after 9 beats,** one of the dashed lines).”

The text above (lines 445-446) reads

“Instead of the direct approach, we have evaluated the fitness function after a short run (9 stimulations at each PCL).”

Line 463: "We suggest" isn't great wording. The section later goes on to say things like "we did not include potassium concentrations..." so why not stick to that sort of language? "We included the initial intracellular sodium..."

The corresponding text was removed from the new version of the manuscript. Thank you.

Line 463: The whole first half of this paragraph feels far more like methods than results. The authors talk about an extra modification made to the GA (intracellular ion concentration initial conditions also allowed to vary as part of the GA), but results without this modification are not shown. If results without were shown, and then commented upon, informing this modification that then led to improved results, I could understand putting this content in the Results section. As it is though, the easiest fix seems to be just to move it into the Methods (which also avoids having to generate any new Results).

Thank you, the text was moved to the lines 258-263.

Line 504: "Consequently, when synthetic AP was used as input data..." This makes it sound like the authors were using something else previously, then switched to synthetic AP data. I know that's not the case, but I just mean this wording is very awkward. Rephrase as something like:
"We observed more robust algorithm convergence in the case of Cauchy mutation, for model fitting to synthetic AP data (Fig. 6B)"

Thank you, the text was modified according to the recommendations. Please see lines 496-497 in the new revision.

Line 521: "a" and "spoil" are in the wrong order.

Thank you, the error was fixed.

(Already commented above as a general point there, but kept here for posterity.)
Line 666: Why are the other patients, 3-7 only having their APs displayed to indicate the extent and character of the variability, as opposed to patient 2 which features explicit comparison between the data and the CAGE-weighted prediction? This feels very arbitrary, essentially like the results worked well for patient 2 and not for any of the other patients. Honestly including patients 3-7 but not properly including them only makes this whole part feel weaker. Does the concept of adjusting the GA-tuned parameters according to CAGE fail for those patients, or was it just not performed for some reason? Is it that APs were not actually measured, only CAGE data, so this is the best that can be done? If so, make this super clear! If it's in here already and I just missed it (possible!), emphasise it, or otherwise, make sure to mention this! Otherwise, the extra patient data here just fills my mind with more questions, as opposed to strengthening the result.

We thank the reviewer for pointing this out, as mentioned above, the Methods section was changed accordingly to clarify this issue. Moreover, short explanation was added to the lines 664-666:

“As noted above, functional data was not available for these particular patients, but the variability between the models is within physiological range [11].”

(Already commented above as a general point there, but kept here for posterity.)
Line 685: Not sure if it's just the version I have on .pdf as a reviewer, but Figure 11 (one of the most important figures!) does not look great. There are serious image artifacts and it's actually a struggle for me to make out the lines plotting the AP curves against the data in some cases. The red and blue lines often blur in with one another and I do not have colourblindness, it is simply a result of the artifacting, plus their thinness. Much of this might just be limited to the version I'm looking at, but please see if this can be improved.

As we answered above, we think that the only solution of the problem is downloading high resolution figures using the link, but we are not sure if the link from PDF document works for reviewers. We have double-checked that the resolution of the figure is high and the high resolution images are available for download to us.

Line 705: In contrast to the comment for line 666 the treatment of patients 8-14 is far more transparent. The successes and failures of the approach are shown clearly, there's no hiding. This makes me feel a lot more comfortable, and the fact that the method works at all (even if not always) is still really nice. Seeing changes in DNA map directly through to changes in parameters that actually do a decent job of predicting the patient's AP is a HUGE point in favour of the underlying AP model doing the right thing (something which is always a little in question because there's many competing models that all give different predictions). "which indirectly indicates GA-output parameters precision for this particular case" is seriously understating what is actually a really cool result! I think this part needs way more/better discussion, including both a better emphasis on the good result,and some more insight into why the authors believe other cases may have failed. I know that some thoughts along these lines are given for why the patient 11-based models weren't great, but another point that could be made is that the models always seem to overestimate the APD when they fail to fit the data super well. If the authors have any thinking as to why, that'd be nice to add. Also, the models based on patient 8 and patient 9 seem very similar for most of the patients, but as pointed out, the parameter values themselves are very different. Worth commenting on that everpresent issue of multiple parameter sets giving similar behaviours, and that actually you've found two sets of parameters that also behave pretty much the same across different pacing frequencies.

We thank the reviewer for positive evaluation of our results. We do hope that the new discussion is more comprehensive and impactful. Several clarifications were added to the Results section, but, most importantly, new lines 865-889 read:

“This heterogeneity also explains why models shown in Fig. 10 tend to overestimate the APD. In order to exclude possible effect of ATP-dependent shortening of AP, we have manually chosen the recording with the longest AP as GA-input. On the other hand, probable local tissue damage at the site of recording might result in an opposite effect, *i.e.* downregulation of K-channels [48] prolonging the APD.

In the Fig. 10 and Table 5 we also demonstrate that minor changes in AP waveform may result in major changes of parameters values. Despite the differences in *Patients 9-11*-based models, in some cases the AP waveforms and restitution were very similar. Consequently, this imposes a limitation on the GA-output model: it has to follow experimental data very closely and perturbations of experimental AP waveform are likely to spoil algorithm performance. In our opinion, this fact also implies that the modifications we introduced to GA were crucial to the successful gene expression-based prediction of AP waveform.

As was noted previously [49] modern cardiac models coalesce multiple studies performed on different species, using different experimental conditions. Moreover, given the complexity of modern models, it is possible to describe particular dataset using different parameters [8-10] (see also Table 5 and Fig. 10). These facts leave us with questions: even if the model describes the particular dataset underlying it, what is the predictive capability of computer simulations? Is it possible to extrapolate model predictions to different clinical or experimental conditions? For example, it is possible that the model description of a particular ionic current is imprecise, but other model components are instead tuned to counterbalance this imprecision. In this case, the model components rescaling would most probably upset the balance and result in model failure to predict AP waveform. In this study, we have shown that it is possible to use computational model to map expression profile to cardiac function and predict actual AP waveform (Figs 9-11). This fact makes a point not only in favor of particular GA-output parameters set, but also indicates the underlying computational model precision. ”

Lines 895-899:

“Another possible implication of the provided technique is drug effects investigation. For example, ionic channel blockers often affect several ionic currents. Using GA to measure the effects of a drug provides a cheaper way to recover several ionic currents dose-response curve simultaneously then the patch-clamp technique.”

Lines 747-752 in the result section:

“It is also interesting that despite major differences in parameters, models behavior was surprisingly similar not only in the case of *Patient 9*, but also in a number of other cases. At least partially, this could be explained by similarities in expression profiles: for example, differences between *Patient 11* and *Patient 13* genes expression corresponding to I_Kr_, I_Ks_, I_to_, I_CaL_, RyR and CaMKII were less than 9 % (see Table C in S1 text). ”

Lines 908-912 in the Limitations section:

“Patient 11-model appears to underestimate the sodium current and consequently Patient 11-based models failed to depolarize in half of the cases. Patient 9-based model seem to overestimate APD and, consequently, in half of the cases it was impossible to pace the model at the fastest frequency (Fig. 10). This implies the imprecision of Patients 9 and 11 GA-output parameter values.”

Line 738: "The difference in APD80 is below 15ms for CL 1000ms". So what? Why pick out one biomarker, at one pacing frequency, and only talk about it? Is this supposed to be picking out the biggest point of discrepancy between the two curves, because otherwise their agreement is very good? If so, please communicate this (and also feel to use stronger words than "relatively close" on line 736, because although it's hard to make out on the low-quality figure, I'd call them a lot more than "relatively close" if I'm seeing them correctly!)

Thank you, we have removed the sentence and instead inserted a portion of text that, we believe, communicates the idea better:

“Surprisingly, in this case, the GA-output model actually performed worse than the mRNA-based model, the difference is most prominent at 250 ms PCL: in the former case (*Patient 9*-based model) APD80 error was 26 ms, in the latter (*Patient 8*-based model) APD80 error was 3 ms. On the other hand, this difference resulted only in slight RMSE_250ms_ difference: 5.7 vs 5.0 mV correspondingly. This implies high sensitivity of GA to AP perturbations that was shown above by noise sensitivity analysis: GA-output model should follow experimental AP waveform closely and minor experimental artefacts might cause major change in parameter values”

Line 745: Table 5 was an enigma to me before multiple re-reads. Are these multipliers? Compared to what baseline? The text says that RyR is 1.5-3.5 times higher for the Patient 8 based model for Patient 9 (as compared to the direct GA fit to patient 9 data) and yet the RyR values are 0.690 and 0.825, respectively, so this is not true! The caption for Table 5 really needs more information, and/or the text does. A reword would help make it so much more understable, too.
For example, if I'm indeed understanding it right, something like: "Table 5: Parameter values (relative to baseline) selected for models for Patient 9 derived via GA, or via comparison of mRNA levels to reference patient". And then the headings are essentially what you have, but make it clear that those are the reference patient. As it is, it's too easy for me to glance at the table and think the first column is results for Patient 8, and the last column for Patient 10. When it's not, all columns show results for Patient 9, but with models derived in different ways (as the caption tries to say, but I feel my wording just provided achieves this much better).

We thank the reviewer for pointing the issue out; the Table 5 was very hard to understand indeed. Regarding the RyR, we apologize, because it is an error on our side. Explanation in the text was modified. Lines 728-730: “Table 5 lists the parameter values for three variants of *Patient 9* model: GA-output model, and two transcription profile-based models.”

Table 5 caption:

“**Table 5: Parameter values (relative to baseline O’Hara-Rudy model [15]) of *Patient 9* models derived via GA or via comparison of mRNA levels to reference patient.**”

The headings also mention “Reference patient” now.

Line 748: The opening sentence of the discussion is very disagreeable to me. The paper hasn't really introduced a GA-based technique that allows personalization of AP models using their dependence on the pacing rate, despite claiming this. Refs [2,5] in the paper are already credited earlier in the paper as doing that. What this paper *does* do is bring improvements to the methodology. And then the CAGE and RNA-seq stuff, which is really striking. The discussion should tie these results together. "Look, our GA fits using our new improved methodology are so good, that we can even go and scale them based on DNA/RNA readings and go and recapture intersubject variability!" Perhaps that claim is a little strong, as poorer fits from older methods might still achieve much the same and this wasn't explored, but I think that is the direction you should be phrasing things in.

Thank you. We agree. We have revised the opening sentence and added a few sentences here:

“In this study, firstly, we have introduced a novel GA modification allowing one to personalize cardiac electrophysiology models using steady-state AP recordings dependence on PCL as input data. The algorithm modification is based on the idea that parametric optimization and slow variables steady state search should be performed simultaneously for effective convergence. Secondly, we have tested the modifications we introduced on synthetic action potentials proving our modifications to be advantageous for overall algorithm performance. Finally, we have tested the algorithm performance against cardiac optical mapping experimental data and mRNA expression profile. The output parameters precision was confirmed by the observation that mRNA-based models predict patients AP waveform and restitution. Essentially, a combination of GA with the mRNA expression measurements provides a novel technique for model personalization.”

Line 770: "This approach has two disadvantages:" is not the way this should be worded. Why not instead something like "There remained two issues to be addressed:". The two things that are discussed are not so much disadvantages of your approach, as just challenges for any method trying to find steady state quickly insides of a GA (or other optimization method). So my wording here takes out the unnecessary badmouthing of your method.

Thank you for pointing out this unclear text in our manuscript. Actually, we intended, unsuccessfully, to discuss and justify the mutation of slow variables. We revised the text to express it hopefully more clearly. Please see lines 782-788:

“Without further modifications this approach has two limitations:… In order to address these issues, two slow variables: intracellular sodium concentration and sarcoplasmic reticulum calcium load are treated as model parameters by the algorithm”.

Line 869: Presuming I'm interpreting the figure right (low quality makes it hard to see), I think the Patient 9-based model did a much better job than the authors give it credit for, here. Patient 8 did the best, but Patient 9 actually fits well to more patient+pacing rate combinations than it fails to fit to. This is pretty good performance for a parameter tuning that is coming from indirect measurements, and as I mentioned earlier, actually has implications regarding how much confidence to give our AP model predictions (in a good way!). I don't think it should just be lumped in with the Patient 11-based model as "failing to fit".

We thank the reviewer for this suggestion. However, we do believe Patient 9 and Patient 11 models to be relatively imprecise. The text was modified to communicate this message:

“Patient 11-model appears to underestimate the sodium current and consequently Patient 11-based models failed to depolarize in half of the cases. Patient 9-based model seem to overestimate APD and, consequently, in half of the cases it was impossible to pace the model at the fastest frequency (Fig. 10). This implies the imprecision of Patients 9 and 11 GA-output parameter values.”

Specific Comments (Less Important / Positives)

-----------------------------------------------------------------------------------------------------

Lines 93-133: This was a big improvement over the previous version. Well written.

Line 168: Figure 2 is much improved!

Thank you!

Line 181: The explanation starting here was a bit hard to understand. Not a major point but I think it could be expressed more clearly.

We hope that new text is clearer:

“The algorithm is optimized for optical AP recordings, where absolute transmembrane potential (TP) values are not known, and thus input data (both synthetic and experimental) is renormalized by the algorithm prior to every fitness function calculation. The following technique was used for renormalization. Firstly, input AP waveform is shifted along the time axis in order to superimpose compared waveforms; in particular, half-maximum depolarization of the waveforms to be compared is used as a reference point. After that, input AP is rescaled: $V_{rescaled}=\alpha V+\beta$**,** where $\alpha$ and $\beta$ coefficients are determined by the least-squares technique to minimize the deviation between input and output AP.”

Line 268: Maybe put the i's and NSR's on these concentrations, though I appreciate it's uglier when you also want to use the subscript to mark the pacing frequency. Not a major issue to me either way.
We have added the subscripts.

Line 286: I agree with the other reviewer's first round comments, I think that the principal components are a much nicer way to visualise this!

Thank you!

Line 302: The point is labelled here R, and another later A, but then R and A are not referred to again from what I saw. Labelling them like this just made it more confusing to me, (x0,y0) and (xc,yc) would be fine.

R and A were removed from the text.

Line 316: Figure 5 could probably be moved to the supplement, in my opinion. The point that large numbers of elite particles performs better is already made in Figure 4.
We have shifted the figure to the supplement.

Line 456: This is a nice explanation of why the method presented in the paper could be expected to work. I feel like it fits better where the method is presented, instead of in results, but this isn't major.
Thank you, we have shifted the text partially, but the text below also provides the proof that output variables are steady-state indeed, thus we believe that the manuscript is more comprehensible with the explanation in the results section.

Line 584: The section "Input data requirements" talks first about the amount of restitution information required for different parameters to be correctly identified, and then the requirements in terms of noise. I would actually just break this up into two separate sections, with more informative headings on each. "Input data requirements" doesn't immediately make me think of restitution/requirements on number of pacing frequencies. Also, it seems to me like other target values for the parameters could likely change how many different frequencies are required to identify those different parameters. So talking about this like a requirement on the input data has been identified feels a little wrong.

Line 604: On the other hand, while it might be true that different APs also showed a different sensitivity to noise, I'm far more comfortable with this section being expressed in terms of the "required signal-to-noise ratio". I also appreciate its inclusion, it was interesting and I think it's valuable.

We have divided the text in two subsections (“Input data requirements: restitution information.” and “Input data requirements: signal-to-noise ratio.”). Indeed changing the balance between ionic currents considerably (for example increasing IKs, while decreasing IKr) would affect the algorithm sensitivity to the amount of restitution information. We have acknowledged it in the former subsection, lines 587-590:

“While using considerably different target parameter values would affect the results shown in this section, it provides an estimate of the amount of restitution information required to determine identifiable parameters. Consequently, we have recorded the AP waveform at 4 PCLs in the experiments described below.”
